# Serotoninergic and dopaminergic modulation of cortico-striatal circuit in executive and attention deficits induced by NMDA receptor hypofunction in the 5-choice serial reaction time task

**DOI:** 10.3389/fncir.2014.00058

**Published:** 2014-06-11

**Authors:** Mirjana Carli, Roberto W. Invernizzi

**Affiliations:** Laboratory of Neurochemistry and Behavior, Department of Neuroscience, IRCCS-Istituto di Ricerche Farmacologiche “Mario Negri”Milano, Italy

**Keywords:** 5-HT receptors, DA receptors, NMDA receptor, PFC, dorsal striatum, attention, executive functions, GLU release

## Abstract

Executive functions are an emerging propriety of neuronal processing in circuits encompassing frontal cortex and other cortical and subcortical brain regions such as basal ganglia and thalamus. Glutamate serves as the major neurotrasmitter in these circuits where glutamate receptors of NMDA type play key role. Serotonin and dopamine afferents are in position to modulate intrinsic glutamate neurotransmission along these circuits and in turn to optimize circuit performance for specific aspects of executive control over behavior. In this review, we focus on the 5-choice serial reaction time task which is able to provide various measures of attention and executive control over performance in rodents and the ability of prefrontocortical and striatal serotonin 5-HT_1A_, 5-HT_2A_, and 5-HT_2C_ as well as dopamine D_1_- and D_2_-like receptors to modulate different aspects of executive and attention disturbances induced by NMDA receptor hypofunction in the prefrontal cortex. These behavioral studies are integrated with findings from microdialysis studies. These studies illustrate the control of attention selectivity by serotonin 5-HT_1A_, 5-HT_2A_, 5-HT_2C_, and dopamine D_1_- but not D_2_-like receptors and a distinct contribution of these cortical and striatal serotonin and dopamine receptors to the control of different aspects of executive control over performance such as impulsivity and compulsivity. An association between NMDA antagonist-induced increase in glutamate release in the prefrontal cortex and attention is suggested. Collectively, this review highlights the functional interaction of serotonin and dopamine with NMDA dependent glutamate neurotransmission in the cortico-striatal circuitry for specific cognitive demands and may shed some light on how dysregulation of neuronal processing in these circuits may be implicated in specific neuropsychiatric disorders.

## Introduction

The integrated activity across frontal cortex and other cortical and sub-cortical brain regions supports a number of cognitive processes subsumed under the term “executive function.” These cognitive processes comprise: selective allocation of attentional resources, maintenance, retrieval, and manipulation of information in working memory, formulation and planning of appropriate sequences of actions, inhibition of inappropriate responses and decision-making on the basis of positive or negative outcomes. Neuropsychological evidence has suggested that executive functioning is critically dependent on the frontal cortex (Fuster, 2009) and indeed the term executive function and frontal lobe function have often been used interchangeably. However, numerous studies in healthy human subjects, monkeys, and rats are suggesting that executive processes are not an exclusive property of frontal cortex but that are mediated by networks incorporating multiple cortical regions (posterior/parietal and prefrontal) as well as cortico-striatal-thalamic circuitry linking regions of the frontal cortex via basal ganglia to the thalamus. The executive dysfunctions associated with basal ganglia disorders have provided further evidence that fronto-striatal circuitry rather than discrete frontal regions may be important in mediating these functions.

The neural activity in the cortico-striatal circuitry is modulated by a diversity of neurochemical influences, each contributing to its functional integrity in a specific manner. Glutamate serves as the major excitatory neurotransmitter in the brain. Given the multiplicity of its receptor subtypes, a particular neuron's response to glutamate is determined by the presence and organization of diverse receptor subtypes; ionotropic N-methyl D-aspartate (NMDA), AMPA and kainate and metabotropic mGlu receptors. The NMDA receptors are especially interesting as various studies show that they are able to support persistent firing of cortical neurons (Compte et al., 2000; Wang, 2013). Evidence drawn from studies with rodents, monkeys and humans using multidisciplinary approaches have suggested that neuronal signaling via glutamatergic NMDA receptors play a central role in prefrontal cortex (PFC) activity and its cognitive functions such as working memory, attention, reversal learning (Malhotra et al., 1996; Moghaddam and Adams, 1998; Honey et al., 2003, 2004; Amitai and Markou, 2010; Neill et al., 2010; Arnsten et al., 2012; Pehrson et al., 2013; Wang et al., 2013).

The cortico-striatal circuitry receives innervations from all of the major ascending neurotransmitter systems, which include dopamine (DA), noradrenaline (NE), serotonin (5-HT), and acetycholine (ACh). Notably, studies manipulating the activity of the ascending neurotransmitter systems have demonstrated a rather selective role of these neuromodulatory systems in executive functions (Robbins, 2013). DA appears to play a role in stabilization of representations in processes such as working memory and attention control while NE contribute by enhancing the signal in cognitive operation of the PFC. The 5-HT has been shown to contribute in some of the processes implicated in the cognitive flexibility and impulsivity. The ACh innervation of the PFC has been implicated in attention and spatial working memory. Among these neuromodulatory pathways DA and 5-HT have received special attention for their putative involvement in the pathophysiology of neuropsychiatric disorders such as for example schizophrenia where cognitive functioning is an important indicator of outcome (Green et al., 2004; Lewis, 2004; Gold et al., 2007; Luck and Gold, 2008).

The overlap and convergence of DAergic and 5-HTergic forebrain projections with glutamatergic projections provide a framework for a complex neuronal interaction, which could support various cognitive functions. Underlying the complexity of DA- and 5-HT-glutamate interaction is the co-localization of DA and 5-HT receptors with glutamate receptors within cortico-striatal circuitry. Thus, it is apparent that specific components of executive functions may be the results of convergence points between NMDA receptor signaling and the activity in these neuromodulatory systems. The two classes the DA receptors D_1_-like (D_1_ and D_5_) and D_2_-like (D_2_, D_3_ and D_4_) all belong to G-protein coupled receptors (GPCR); the D_1_-like receptors couple to the stimulatory Gs protein while D_2_-like couple to the inhibitory Gi/Go protein. So far seven families of serotonin receptors have been identified each with numerous subtypes. With the exception of 5-HT_3_ receptor, a ligand-gated ion channel the remaining receptors belong to the superfamily of GPCR. The electrophysiological, biochemical and behavioral characteristics of the interaction DA/NMDA and 5-HT/NMDA receptors have been studied and have been reviewed extensively (Aghajanian and Marek, 2000; David et al., 2005; Castner and Williams, 2007; Tritsch and Sabatini, 2012; Celada et al., 2013; de Bartolomeis et al., 2013).

Here we will first discuss the role of prefrontocortical NMDA receptors in attention and executive control and in cortico-striatal activity. Next, we will review a series of our systematic studies comparing the performance of animals after pharmacological manipulation of DA and 5-HT receptors activity locally in the medial PFC (mPFC) or in the dorsomedial striatum (dm-STR) in animals in which glutamatergic activity was perturbed by blockade of NMDA receptor in the PFC in a task that entails selective attention and tight organization of a complex response sequence for optimal performance (Carli et al., 1983; Robbins, 2002) and which engages fronto-striatal-thalamic circuitry (Christakou et al., 2001; Chudasama and Muir, 2001; Rogers et al., 2001; Chudasama et al., 2003a). Finally, we will illustrate our findings that suggest an association between NMDA receptor antagonist induced increase in glutamate release and attention deficit.

## NMDA receptors in the PFC, attention, executive control, and cortico-striatal activation

Attention allows the subject to engage with its environment by selecting information relevant for its behavior. The relevant information is selected by top-down modulation of neural activity in posterior cortical areas by signals arising from the PFC (Buschman and Miller, 2007; Saalmann et al., 2007; Noudoost et al., 2010). Various lines of evidence demonstrate that persistent firing of pyramidal cells not only support working memory (Funahashi et al., 1989; Wang et al., 2013) but it contribute also to the process of attentional selection (Lebedev et al., 2004). Activation of NMDA receptors on local recurrent synapses rather than AMPA receptor stimulation has been shown to support persistent neuronal activity within the mPFC during the delay period in a working memory task (Wang et al., 2013) but their contribution to attention-induced firing is unclear. However, the attention-driven improvements in signal stability and noise correlation in the macaque visual cortex (area V1) has been shown to depend on high NMDA/AMPA receptor ratio (Herrero et al., 2013).

In our studies in rats we have focused on the control of attention, specifically on the process of input selection; the selection of task-relevant inputs for further processing (Luck and Gold, 2008; Lustig et al., 2013). This aspect of attention is somewhat distinguished from that where the attention is put on the selective activation and maintenance of task-appropriate rules (Luck and Gold, 2008; Gilmour et al., 2013). The most common experimental paradigms used for examining input selection processes of attention are continuous performance tasks among, which is the 5-choice serial reaction time (5-CSRT) task (Lustig et al., 2013). As in most cognitive task the successful performance requires the contribution of several factors other than control of attention and may thus tap as well on executive control processes.

### 5-choice serial reaction time task

**Figure 1:**
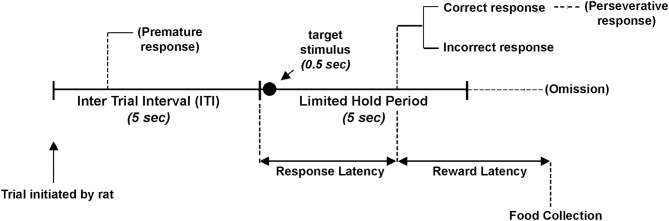
**Schematic diagram of the 5-choice serial reaction time task**. After a waiting period of 5 s (ITI) a target stimulus is presented for 0.5 s in one of the five apertures in a random order. The rat is allowed to make its choice to respond correctly or incorrectly. After a correct nose-poke in one of the apertures rat is rewarded with a food pellet. An incorrect nose-poke or a failure to respond within the 5 s of stimulus onset (limited hold) is followed by 5 s of darkness (time-out) and no food. Responses made during the ITI and those made after a correct or incorrect response are followed by time-out.

For rats (Carli et al., 1983) (Figure 1) the requirement during the 5-CSRT task performance is to sustain spatial attention divided among five locations to detect a brief visual stimulus over a large number of trials. Performance is characterized in terms of accuracy of visual discrimination, omissions, speed of responding and by different aspects of executive control such as premature and perseverative responses (see Robbins, 2002 for a detailed description and discussion of these performance measures). The main measure of the selective spatial attention in the 5-CSRT task is accuracy of visual discrimination. Correct responses are rewarded by a food pellet while incorrect responses or failure to respond within the allotted time (omission) result in few seconds of darkness (time out period). Accuracy is independent of omissions and it is relatively impervious to potential confounds such as changes in motor activity or motivation (see Robbins, 2002). Premature responses that occur before the onset of visual stimulus may arise as a consequence of animal not being able to wait for a reward related cue. These “impulsive” responses measure an aspect of response inhibition that is related to response selection but also to action restraint during waiting and could be considered a type of motor impulsivity (Evenden, 1999; Dalley et al., 2011). Nose poke responses after the correct target detection has been performed are defined as perseverative responses and are considered an indicator of “compulsivity.” Perseverative responses constitute persistence in an initially rewarded behavior such as nose poke (even though is no longer rewarded) and may be regarded as inability to alter behavior in reaction to changing task demands thus representing a measure of behavioral flexibility. Premature and perseverative response result in time out. Responses during time out are not reported usually even if they may constitute an additional parameter reflecting compulsivity (Amitai and Markou, 2010). Finally, a measure of response latency (i.e., mean latency to make a correct response) likely reflects decision time as long as changes in motivation and motor status are ruled out.

## NMDA receptors in the mPFC and 5-CSRT task performance

The NMDA glutamate receptor is a ligand-gated ion channel composed of multiple subunits, which responds rapidly to glutamate by conducting cation currents that depolarize neurons rapidly. In the cerebral cortex NMDA receptors are preferentially expressed by pyramidal neurons particularly in layers II, III, V, and VI but also in excitatory and inhibitory axon terminals (Conti et al., 1997) particularly on parvalbumine (PV+) labeled GABA interneurons (Huntley et al., 1994). Changes in cortical NMDA transmission have consequences for other neurotransmitters locally (for example GABA) and distally (for example DA and 5-HT).

The selective blockade of NMDA receptors located in the mPFC by a competitive NMDA receptor antagonist 3-(R)-2-carboxypiperazin-4-propyl-1-phosphonic acid (R-CPP) has a profound impact on rats' performance in the 5-CSRT task (Table 1). The performance impairment is characterized by deficit in accuracy, increased omissions and correct response latency and by a concomitant loss of executive control in the form of increased premature and perseverative responses. These effects are robust (about 20% decrease in accuracy, while the number of premature and perseverative responses are increased by 2 to 3-fold) and consistent across many independent experiments. This pattern of effects resembles to that after lesions of the mPFC (Muir et al., 1996; Passetti et al., 2002) and clearly implicates NMDA receptor signaling in the mPFC for the successful performance of the task.

**Table 1:** **Effects of blockade of frontocortical NMDA receptors on attentional performance**.

	**mPFC^a^**	**PrL PFC^b^**	**InF PFC^b^**	**ACC^c^**
Accuracy	↓	↓	↓	0
Omissions	↑	↑	↑	↑
Premature	↑	0	↑	0
Perseverative	↑	0	0	nr
Latency correct	↑	↑	↑	nr
Latency reward	0	↑	↑	nr

Data from:

a *Mirjana et al., 2004*,

b *Murphy et al., 2005*,

c *Pehrson et al., 2013*.

*mPFC, medial prefrontal cortex; PrL PFC, prelimbic prefrontal cortex; InF PFC, infralimbic prefrontal cortex; ACC, anterior cingulate cortex*.

↓, *decrease;* ↑, *increase;* 0, *no effect; nr, not reported*

The effects of systemic administration of non-competitive NMDA receptor antagonists such as phencyclidine (PCP), dizocilpine and ketamine on rats' performance in the 5-CSRT task appears to be highly dependent on the type of treatment regimen used. First exposure to these drugs often leads to non-specific effects in some animals such as ataxia and head weaving which are incompatible with the performance of this task while after repeated exposures these effects subside and rats start to show the characteristic deficit in performance; decreased accuracy and increased impulsivity and compulsivity (Grottick and Higgins, 2000; Higgins et al., 2003b; Le Pen et al., 2003; Amitai et al., 2007; Auclair et al., 2009; Amitai and Markou, 2010; Smith et al., 2011). In contrast rats tested after a wash-out period from sub-chronic PCP treatment do not show any performance deficit in the 5-CSRT task. However, Barnes et al. (2012b) using a 5-choice continuous performance task (5C-CPT), which is a version of the 5CSRT task specifically designed to add non-targets to which the subject must inhibit responding, were able to show an attention/vigilance deficit but only when the attentional load was increased.

Attentional impairment may almost certainly account for accuracy deficits observed in this task after injections of R-CPP (10–50 ng/side). However, the accuracy of rats in this task depends also on temporal organization of behavior, as responses initiated late are more likely to be incorrect. Naïve rats make the majority of nose poke responses in the holes (about 80% almost all correct) in a narrow time window (0–0.8 s) of stimulus presentation (Passetti et al., 2002). In analogy to what reported for mPFC lesioned rats it could not be excluded that the temporal distribution of responses of R-CPP-injected rats was more random across a much larger time window thus suggesting that they are “distracted/disorganized” (Passetti et al., 2002). A commission error in the 5-CSRT task may be the result of a faulty decision process, distraction or inability to hold “on-line” the planned response. Thus, it could not be excluded that additional deficit in response selection, increased distractability/disorganization and working memory may account for accuracy deficit after R-CPP. The impaired response selection is an important component of attentional deficit and the correct response latency may reflect the speed of processing involved in the input selection mechanisms of attention and in operations of decisional processes in response selection or both. Since correct and incorrect responses in this task have the same motor requirements the slowing of correct but not incorrect responses after R-CPP rule out motor impairment and could suggest the slowing of input selection processing speed. However, dysfunctional mechanisms of stimulus detection most likely due to distraction or temporal disorganization may certainly contribute. Thus, it could be argued that on occasions when the animals were able to overcome “distraction” and respond correctly they were doing it at the cost of slower responding. This indicates that animals injected with R-CPP when they correctly detected the visual stimulus could hold “on-line” mental representation of planned responses well after the visual stimulus has disappeared. In line with this suggestion are observations that control animals will compensate for the decreased salience of the visual stimulus by increasing the correct response latencies (Carli, 2006b). That deficits in working memory may not completely account for accuracy impairments may also be suggested by recent findings of Chudasama et al. (2005) who using an attention-working memory combined task have shown that rats with PFC lesions were impaired on the attentional but not on the working memory component of the task.

In addition to accuracy deficit R-CPP-injected rats made more omissions. This may suggest that rats did not orient their attention on the stimulus presentation array in time or were engaging in some other behavior thus missing the stimulus presentation. The accuracy and omission deficit were completely abolished by prolonging the stimulus duration (see Figure 3 in Mirjana et al., 2004). Because the frequency of stimulus presentation was regular relative to each trial initiation, when the stimulus duration was increased, the position of the visual target in both space and time was emphasized, thus facilitating accurate responding. The mean latency to collect the earned reward, which represents an additional measure of motivation and/or motor function, was not affected by R-CPP. Together, these findings rule out the possibility that the R-CPP-induced impairments in accuracy and omissions were a consequence of hyperactivity, poor motivation or a failure to make associations or remember the general rules of the task.

Impulsivity and perseveration are both intimately related to executive attentional processes that enable accurate response selection in the face of distraction and interference (Shallice, 1982; Robbins, 1996). Increasing the duration of the target stimulus reduces while decreasing it increases premature and perseverative responses suggesting that premature and perseverative responses in the 5-CSRT task may be under attentional control (Christakou et al., 2001; Carli, 2006b). However, the R-CPP-induced increase in anticipatory and perseverative responses persisted even when the longer stimulus helped alleviate the accuracy and omissions deficits (see Figure 3 in Mirjana et al., 2004). It may be argued that there was a primary deficit of response inhibition making the animals “impulsive” and “compulsive.” Increased impulsivity in this task has been reported after highly arousing stimuli such as brief presentation of loud white noise during the waiting period (Carli et al., 1983), which may lead to attentional deficit (Carli et al., 1983). The inverted U-shaped function linking arousal and performance (Yerkes and Dodson, 1908) has been shown in human subjects performing a 5-CSRT task under conditions of elevated arousal (Wilkinson, 1963). The hypo-function of NMDA receptors in the mPFC may thus lead to a behavioral profile compatible with a state of hyper-arousal. A possible contribution of NMDA antagonist-induced NE release (Lena et al., 2007) to the state of hyper-arousal and consequent impairment in attention cannot be excluded as high levels of tonic NE activity is associated with an inability to focus attention (Aston-Jones et al., 2000).

The increased perseverative responding, which is in line with that reported after excitotoxic lesions of the mPFC (Muir et al., 1996) could be the result of R-CPP preventing the suppression of responses once effective for obtaining reward. The perseverative deficit was not general; it was specifically directed to the stimulus array holes and not the panel of the food magazine.

Evidence for functional heterogeneity of rat PFC and NMDA receptors therein were shown by Passetti et al. (2002) and Chudasama et al. (2003b) who reported that impairments in attentional accuracy after lesions to the mPFC (Muir et al., 1996; Passetti et al., 2002) are mainly reproduced by lesions confined to more dorsal (Cg1) aspects of PFC sparing prelimbic (PrL) and infralimbic (InF) sub-regions. However, attentional deficit induced by R-CPP injections confined to PrL or InF was less well localized (Murphy et al., 2005). A recent study comparing systemic and local application of dizocilpine an NMDA antagonist into the anterior cingulate cortex (ACC) (area Cg1) in rats performing a 3-choice version of the task report that while systemic administration of dizocilpine affected accuracy and omissions local application increased omissions without concomitant changes in accuracy. This finding would suggest separable roles for NMDA receptor in the PFC and ACC for the control of attention (Pehrson et al., 2013). The PrL sub-region of PFC has been shown to be particularly involved in perseverative responding (Passetti et al., 2002; Chudasama et al., 2003b) whereas lesions or blockade of NMDA receptors in the InF sub-region mainly affect premature “impulsive” responding (Chudasama et al., 2003b; Murphy et al., 2005). However, in the study by Murphy et al. (2005) perseverative responses were not affected by blockade of NMDA receptors in the PrL. The failure to see changes in this behavior may reflect the fact that in contrast to studies in which perseverative errors are followed by darkness and time-out (Passetti et al., 2002; Chudasama et al., 2003b; Mirjana et al., 2004) in the study by Murphy et al. (2005) they had no consequences. This may suggest that NMDA receptors are implicated in the control of those behaviors that are relevant for the success and not those of no-consequences.

### NMDA receptors in the mPFC and cortico-striatal activation

Numerous studies in rodents show that acute or repeated administration of NMDA antagonists such as PCP, dizocilpine and ketamine consistently lead to disinhibition of the firing of pyramidal neurons (Jackson et al., 2004) most probably by decreasing the activity of GABA interneurons (Homayoun and Moghaddam, 2007) whose response depends on the firing pattern of pyramidal cells (Thomson, 2000; Shi and Zhang, 2003). NMDA receptor antagonists increase glutamate, 5-HT and NE release in the PFC (Moghaddam et al., 1997; Moghaddam and Adams, 1998; Abekawa et al., 2006; Lena et al., 2007; Lopez-Gil et al., 2007). Systemic PCP and dizocilpine also reduce extracellular GABA in the mPFC (Yonezawa et al., 1998) and there is evidence that glutamate release is inhibited by GABA (Pende et al., 1993; Bonanno et al., 1997; Perkinton and Sihra, 1998). Similarly, intra-mPFC infusion of R-CPP to conscious rats increased glutamate efflux within this brain area (Ceglia et al., 2004; Abekawa et al., 2006; Calcagno et al., 2006, 2009) and lowered GABA levels (Calcagno et al., 2009; Agnoli et al., 2013). The glutamate increase elicited by R-CPP is suppressed by TTX added to the medium perfusing the microdialysis probe suggesting that neuronal activity is required. Thus, the effect of R-CPP on extracellular glutamate may be mediated by direct or indirect suppression of cortical GABAergic transmission, which in turn enhances the release of glutamate. Employing dual-probe microdialysis technique we confirmed and extended these finding showing that R-CPP infused in the mPFC raised also extracellular levels of cortical DA whereas in the dm-STR extracellular levels of GABA were increased together with those of glutamate and DA (Agnoli et al., 2013). These data are summarized in Table 2.

**Table 2:** **Effects of R-CPP infused in the mPFC on glutamate (GLU), GABA and dopamine (DA) release in the PFC and dm-STR**.

	**GLU**	**GABA**	**DA**
PFC	↑	↓	↑
dm-STR	↑	↑	↑

*Data from: Ceglia et al., 2004; Calcagno et al., 2006, 2009; Carli et al., 2011a,b; Agnoli et al., 2013*.

↑, *increase;* ↓, *decrease*.

The activation of glutamate neurotransmission in the mPFC and increased firing activity of pyramidal-projecting neurons may drive the increase in endogenous glutamate release in the dm-STR, which in turn may increase GABA and DA release. The NMDA/GABA interaction, regulate DA levels in the PFC and striatum (Balla et al., 2009). Reducing GABA transmission in the mPFC with GABA_A_ receptor antagonist bicuculline or infusion of glutamate increases DA release in the dorsal-STR and these effects are abolished by intracortical infusion of dizocilpine or the GABA_A_ agonist muscimol (Matsumoto et al., 2003). It is conceivable that glutamate by activating NMDA receptors on striatal medium spiny GABA neurons or interneurons facilitates GABA release (Morari et al., 1993, 1994, 1996; Young and Bradford, 1993).

Systemic administration of NMDA receptor antagonists such as ketamine and PCP had no significant effect on extracellular glutamate in the striatum (Lillrank et al., 1994; Moghaddam et al., 1997), and caused no changes or increased basal but inhibited K^+^ evoked GABA release (Lillrank et al., 1994; Hondo et al., 1995). These findings suggest that different NMDA receptor antagonists may have different effects on extracellular glutamate and GABA depending on the route of administration and brain region considered.

These findings in rats are paralleled by data from functional magnetic resonance imaging (fMRI) in human subjects showing that NMDA receptor antagonist such as ketamine at doses that cause specific behavioral impairment in the executive component of a working memory task (Honey et al., 2003), increases BOLD response in a brain system comprising frontal cortex, parietal cortex, putamen, and caudate nucleus (Honey et al., 2004) and increases glutamine, a putative marker of glutamate release (Rowland et al., 2005). Some more recent studies assessing PFC activation and global connectivity within a working memory network during rest or during task performance have reported an increased or decreased ketamine-associated activation, respectively (Driesen et al., 2013a,b).

## Serotonin/NMDA receptors interaction and attention performance

### Cortical 5-HT receptors

The functions of 5-HT are afforded by the concerted actions of multiple 5-HT receptor subtypes and as shown repeatedly 5-HT through its receptor subtypes exert diverse, often antagonistic actions on the same behavioral response. Several lesion and pharmacological studies have attempted to define the role of 5-HT and its various receptors in different aspects of 5-CSRT task (for a review of these studies see Robbins, 2002).

The mPFC receives extensive 5-HT innervation from the dorsal (DR) and median (MR) raphè nuclei and contains several 5-HT receptors, with particular abundance of 5HT_1A_ and 5-HT_2A_ and 5-HT_2C_ subtypes (Azmitia and Segal, 1978; Steinbusch, 1984; Blue et al., 1988; Jakab and Goldman-Rakic, 1998, 2000; Barnes and Sharp, 1999; Clemett et al., 2000; Pandey et al., 2006). In the PFC the 5-HT1A and 5-HT2A receptors are expressed throughout cortical regions with a greater proportion of expression on pyramidal rather than GABA interneurons (Santana et al., 2004). The 5-HT_2C_ receptors are mainly expressed on pyramidal neurons (Clemett et al., 2000; Puig et al., 2010) and not in fast-spiking interneurons (Puig et al., 2010) but another immunohistochemical study using a different antibody shows more than 50% of the 5-HT_2C_ receptors on GABA neurons (Liu et al., 2007). These 5-HT receptors have been extensively characterized in terms of their localization to pyramidal and GABA interneurons as well as biochemically and electrophysiologically and a detailed review of their impact on cortical neuron activity can be found in Celada et al. (2013).

Stimulation of 5-HT_1A_ receptors by 8-OH-DPAT inhibits NMDA-mediated synaptic excitation in the rat visual cortex (Edagawa et al., 1998) and suppresses glutamate signaling in the PFC by reducing NMDA and AMPA receptor currents (Cai et al., 2002). *In vitro* studies show that activation of 5-HT_1A_ receptor reduces NMDA-evoked glutamate release elevation while their blockade has opposite effects (Matsuyama et al., 1996; Maura and Raiteri, 1996). In *in vivo* studies PFC application of 8-OH-DPAT does not affect NMDA-evoked glutamate release, while the 5-HT_1A_ receptor antagonist WAY100135 enhance basal and NMDA-evoked glutamate release in the striatum (Dijk et al., 1995). Additionally, the 5-HT_1A_ partial agonists and full antagonists attenuate working memory deficits as well as psychotomimetic effects induced by NMDA antagonists (Harder and Ridley, 2000; Wedzony et al., 2000). Stimulation of 5-HT_1A_ somatodendritic autoreceptors in the DR or blockade of post-synaptic 5-HT_1A_ receptors in the hippocampus remediate the spatial learning deficit induced by blockade of NMDA receptors (Carli et al., 1998).

Activation of 5-HT_2A_/_2C_ receptors by DOI enhances the firing of pyramidal neurons (Puig et al., 2003) and 5-HT release dependent on activation of AMPA receptors (Martin-Ruiz et al., 2001) and increases glutamate levels in the somatosensory cortex (Scruggs et al., 2000, 2003). Activation of 5-HT_2A_/_2C_ receptors in the PFC modulates GABAA receptor currents (Feng et al., 2001) and increases GABA release (Abi-Saab et al., 1999). Blockade of 5-HT_2A_ receptors reduces NMDA antagonists-induced *fos* expression (Habara et al., 2001), motor hyperactivity (Gleason and Shannon, 1997; Martin et al., 1997, 1998; Swanson and Schoepp, 2002), forced swimming immobility (Corbett et al., 1999) and pre-pulse inhibition (PPI) (Varty et al., 1999). Blockade of 5-HT_2C_ receptors enhances NMDA antagonists-induced motor hyperactivity and DA release (Hutson et al., 2000).

## Performance in the 5-CSRT task

The effects of 5-HT_1A_, 5-HT_2A_, and 5-HT_2C_ receptor agents after systemic or intra-mPFC injections on attention and executive deficits induced by R-CPP (50 ng/sise) injected in the mPFC are summarized in Table 3.

**Table 3:** **Summary of the effects of intra-mPFC R-CPP in combinations with 5-HT_1A_, 5-HT_2A_, and 5-HT_2C_ agents, an mGlu_2/3_ agonist and various antipsychotics on attention and executive control and glutamate (GLU) and serotonin (5-HT) release in the mPFC**.

	**Attention**	**Impulsivity**	**Compulsivity**	**GLU**	**5-HT**
R-CPP^a^^,^^b^	↓	↑	↑	↑	↑
**SEROTONIN AGENTS**					
+ 8-OH-DPAT^c^^,^^d^	0	↑	0	0	0
+ M100907^a^^,^^c^^,^^d^	0	0	↑	0	0
+ Ro60-0175^e^	0	0	↑	0	0
**mGlu_2/3_ AGENTS**					
+ LY379268^f^	0	0	↑	0	nd
**ANTIPSYCHOTICS**					
+ haloperidol^g^^,^^h^	↓	0	0	↑	0
+ aripiprazole^h^	0	↑	0	0	0
+ olanzapine^h^	0	0	↑	0	0
+ clozapine^i^	0	0	↑	0	0
+ sertindole^i^ (0.32 mg/kg)	0	↑	↑	0	0
+ sertindole^i^ (2.5 mg/kg)	↓	↑	↑	↑	↑

Data from:

a *Mirjana et al., 2004*,

b *Ceglia et al., 2004*,

c *Carli et al., 2006a*,

d *Calcagno et al., 2006*,

e *Calcagno et al., 2009*,

f *Pozzi et al., 2011*,

g *Baviera et al., 2008*,

h *Carli et al., 2011b*,

i *Carli et al., 2011a*.

↓, *decrease;* ↑, *increase; 0, reversal of R-CPP-induced effect*.

### Accuracy

The behavioral manifestation of the functional interaction between 5-HT_1A_ and 5-HT_2A_ and 5-HT_2C_ with NMDA receptors in the mPFC is the demonstration that selective agonist at 5-HT_1A_ receptor 8-OH-DPAT and antagonist at 5-HT_2A_ receptor M100907 as well as 5-HT_2C_ receptor agonist Ro60-0175 recovered attentional performance deficit due to blockade of NMDA receptor in the mPFC, albeit in a distinct manner (Mirjana et al., 2004; Carli et al., 2006a; Calcagno et al., 2009). Microinjections of 8-OH-DPAT or M100907 in the mPFC prevent accuracy deficit (Carli et al., 2006a). Clearly, the functional opposition between the two 5-HT receptor subtypes on accuracy suggest that the improvement produced by M100907 and 8-OH-DPAT might reside in their opposite activity on common cellular substrates (Araneda and Andrade, 1991; Ashby et al., 1994; Celada et al., 2013). The 5-HT_1A_ but not 5-HT_2A_ or 5-HT_2C_ receptors appear to be involved in decision processes in this task as 8-OH-DPAT but not M100907 or Ro60-0175 reduced correct response latency and omissions (Mirjana et al., 2004; Carli et al., 2006a; Calcagno et al., 2009). DA system and in particular D_1_ receptor in the PFC and in the dm-STR have been shown to impact decision processes in this task (Granon et al., 2000; Robbins, 2002); (see Table 2 in Agnoli et al., 2013). The fact that 8-OH-DPAT infused in the mPFC increases DA efflux in this cortical region (Sakaue et al., 2000) may at least in part explain its effects on speed and omissions.

Injections of 8-OH-DPAT and M100907 in the mPFC in control rats had no effect on accuracy, which is in contrast to what reported by other studies. The effects of 5-HT_1A_ agonists on accuracy in normal rats performing the task under basal conditions depend on whether the 5-HT_1A_ somatodendritic autoreceptors or post-synaptic receptors are activated. Systemic 8-OH-DPAT impaired accuracy and this effect was abolished by 5,7-dihydroxytryptamine lesion or blockade of 5-HT receptors in the DR by a selective 5-HT_1A_ antagonist WAY100635 (Carli and Samanin, 2000). In contrast, Winstanley et al. (2003a) reports a facilitation of accuracy after systemic or intra-cortical injections of 8-OH-DPAT. The role of 5-HT_2A_ receptors in accuracy is much less clear as systemic M100907 had no effect (Winstanley et al., 2004a) and intra-mPFC injection facilitated accuracy at long but not short stimulus duration (Winstanley et al., 2003a). The 5-HT_2C_ receptor do not appear to control accuracy in normal rats as no effect on accuracy has been reported after 5-HT_2C_ receptor agonists or antagonists (Higgins et al., 2003a; Winstanley et al., 2004a; Fletcher et al., 2007, 2011).

### Impulsivity and compulsivity

In contrast to the effects of 5-HT_2A_ receptor antagonist, which reduced premature responses but not perseverative over-responding either after systemic or intra-cortical injection (Mirjana et al., 2004; Carli et al., 2006a), activation of 5-HT_1A_ receptors in the mPFC had no effect on premature but decreased perseverative over-responding (see Table 3). 5-HT acting on 5-HT_2A_ receptors segregated to apical dendrites of pyramidal neurons (Jakab and Goldman-Rakic, 1998) and to GABA interneurons specialized in the perisomatic inhibition of pyramidal cells (Jakab and Goldman-Rakic, 2000) can affect excitatory input (Aghajanian and Marek, 1997) and by acting on 5-HT_1A_ receptors in the axon hilloc (DeFelipe et al., 2001; Czyrak et al., 2003) can suppress the generation of action potential along the axon and influence the activity in subcortical projection areas. Thus, by finely tuning the complex activity of glutamatergic pyramidal neurons, 5-HT may differently influence distinct aspects of executive control. These results clearly demonstrate the selectivity of executive control processes and indicate that impulsivity and compulsivity may be dissociated by 5-HT_1A_ and 5-HT_2A_ receptor mechanisms in the mPFC.

The effects of systemic M100907 and Ro60-0175 on R-CPP-induced impulsivity (Mirjana et al., 2004; Calcagno et al., 2009) are consistent with studies showing similar effects of these compounds on impulsivity but not compulsivity induced by systemic injections of NMDA antagonists dizocilpine and Ro63-1908 (Higgins et al., 2003b; Fletcher et al., 2011). In contrast the 5-HT_2C_ antagonist SB242084 increased premature responses already in control rats and tended to enhance dozocilpine-induced impulsivity (Higgins et al., 2003b).

Previous studies have suggested that enhanced impulsivity in the 5-CSRT task is associated with increased 5-HT turn-over (Puumala and Sirvio, 1998) and release in the PFC (Dalley et al., 2002) and activation of 5-HT_2A_/_2C_ receptors by DOI (Koskinen et al., 2000). However, global forebrain 5-HT depletion consistently results in enhanced impulsivity (Soubrié, 1986; Harrison et al., 1997; Carli and Samanin, 2000; Mobini et al., 2000). This apparent discrepancy may be resolved by 5-HT exerting inhibitory activity on impulsivity through 5-HT_2C_ but not 5-HT_2A_ receptors since decreasing their activity leads to impulsivity (Higgins et al., 2003b; Winstanley et al., 2004a; Fletcher et al., 2007). This suggestion is further supported by findings that activation of 5-HT_2C_ receptors decreases while their suppression increases premature responding in the 5-CSRT task under various conditions such as when the waiting period is increased (Carli, 2006b; Fletcher et al., 2007) or premature responding is enhanced by NMDA receptor antagonists (Higgins et al., 2003b; Calcagno et al., 2009).

Like systemic NMDA receptor antagonists, intra-mPFC infusion of R-CPP enhances DA release in the mPFC (Table 2) (Moghaddam et al., 1997; Del Arco and Mora, 1999; Feenstra et al., 2002). Increasing DA transmission by d-amphetamine increases perseverative responses in the 5-CSRT task (Baunez and Robbins, 1999). Although microdialysis studies show that 8-OH-DPAT increases DA release in the mPFC (Arborelius et al., 1993; Sakaue et al., 2000) it actually reduces the rise in cortical DA release induced by d-amphetamine, stress and isolation rearing (Rasmusson et al., 1994; Kuroki et al., 1996; Ago et al., 2002) and attenuate d-amphetamine-induced motor activation (Przegalinski and Filip, 1997). The D_2_ receptor antagonist haloperidol also decreases R-CPP-induced perseverative responding (Baviera et al., 2008) (Table 3). It is plausible that 8-OH-DPAT could decrease perseverative responding through its action on DA mechanisms. However, the effects of 8-OH-DPAT were due to activation of 5-HT_1A_ receptors in the mPFC as a selective 5-HT_1A_ antagonist WAY100635 completely blocked the effects of 8-OH-DPAT on accuracy deficit and perseverative responding (Carli et al., 2006a).

### Comparison with mGlu_2/3_ receptors

It is worth noting that the effects of M100907 on R-CPP-induced impairments in 5-CSRT task performance and the increase in glutamate release in the mPFC (see Table 3) are mimicked by mGlu_2/3_ receptor agonist LY379268 (Pozzi et al., 2011). 5-HT-evoked excitatory post-synaptic currents are similarly inhibited by 5-HT_2A_ antagonist M100907 and by mGlu_2/3_ receptor agonists (1S,3S)-ACPD and LY354740 and enhanced by the mGlu_2/3_ antagonist LY341495 (Aghajanian and Marek, 1999, 2000; Marek et al., 2000). Activation of 5-HT_2A_ receptors by DOI or LSD increases excitatory post-synaptic currents and potentials, glutamate release, c-fos in PFC, and induces head-twitch response (Aghajanian and Marek, 2000; Gewirtz and Marek, 2000; Klodzinska et al., 2002; Zhai et al., 2003; Gonzalez-Maeso et al., 2008). All these effects are blocked by 5-HT_2A_ antagonists or by mGlu_2/3_ agonists. This functional analogy may be based in part on anatomical overlap of mGlu_2_ particularly in apical dendrites of lamina V with the riches distribution of 5-HT_2A_ receptors (Blue et al., 1988; Aghajanian and Marek, 1999; Marek et al., 2000, 2001) but may also derive from the mGlu_2_ receptor forming a complex through the specific transmembrane helix with 5-HT_2A_ receptor (Gonzalez-Maeso et al., 2008).

### Dorsal striatal 5-HT receptors

The 5-HT afferents arising mainly in the DR nucleus (Steinbusch, 1984) innervate all components of the basal ganglia circuitry (Lavoie and Parent, 1991). The fact that 5-HT modulates not only DA but also GABA and glutamate neurotransmission in the dorsal striatum and output regions of the basal ganglia (Nicholson and Brotchie, 2002) suggest a 5-HTergic regulation of action selection and motor control (Di Matteo et al., 2008) but little is known about their contribution to cognitive function.

Among the various 5-HT receptor subtypes present within dorsal striatum the 5-HT_2A_ and 5-HT_2C_ receptors are particularly abundant (Barnes and Sharp, 1999). They are equally distributed on medium spiny neurons (MSN) forming the direct striatonigral and the indirect striatopallidal output projections but also on GABA and cholinergic (ACh) interneurons (Ward and Dorsa, 1996; Eberle-Wang et al., 1997). These 5-HT_2_ receptor subtypes play a prominent role in the modulation of striatal DA function (Abdallah et al., 2009; Navailles and De Deurwaerdere, 2011) and excite striatal ACh and fast spiking GABA interneurons (Blomeley and Bracci, 2005, 2009). The 5-HT_2_ receptor antagonists administered within the striatum block DA-mediated oral activity (Plech et al., 1995), synergize D_1_-induced locomotor activity (Bishop and Walker, 2003) and cause retrograde amnesia in rats (Prado-Alcala et al., 2003a,b). Loss of 5-HT_2C_ receptors enhances behavioral sensitivity to D_1_ receptor activation (Abdallah et al., 2009).

## Performance in the 5-CSRT task

The effects of 5-HT_2A_ and 5-HT_2C_ receptor agents injected in the dm-STR on attention and executive deficit induced by R-CPP (50 ng/side) injections in the mPFC are summarized in Table 4.

**Table 4:** **The effects of serotonin and dopamine receptors agents injected in the dm-STR on attention and executive deficits induced by R-CPP injections in the mPFC**.

	**Accuracy**	**Impulsivity**	**Compulsivity**
R-CPP	↓	↑	↑
**+ SEROTONIN AGENTS**			
M100907^a^	0	0	0
Ro60-0175^a^	0	0	0
**+ DOPAMINE AGENTS**			
SCH23390^b^	0	0	↑
haloperidol^b^	↓	0	0

Data from

a *Agnoli and Carli, 2011*,

b *Agnoli et al., 2013*.

*↓, decrease; ↑, increase; 0, reversal of R-CPP-induced effect*.

### Accuracy

Activation of 5-HT_2C_ or blockade of 5-HT_2A_ receptors in the dm-STR reduce accuracy deficit induced by R-CPP. These data concur with the above discussed data showing opposing roles of these receptors on the neurochemical processes that support the 5-CSRT task performance deficits induced by NMDA receptor antagonists. However, cortical 5-HT_2A_ receptors exert a much more effective control over attention as much higher dose of M100907 had to be administered in the dm-STR than in the mPFC to achieve an effect on accuracy. The PFC shows much higher levels of 5-HT_2A_ hybridization signals than the dorsal striatum (Pompeiano et al., 1994) and there is a substantial and reciprocal control of the activity of DR cortical 5-HT neurotransmission by PFC (Hajos et al., 1998, 1999; Celada et al., 2001). This control has an important functional role; for example 5-HT depletion abolishes the facilitatory effects of M100907 on accuracy and its ability to prevent R-CPP-induced glutamate release in the mPFC (Winstanley et al., 2004a; Calcagno et al., 2009) but also in stress-induced activation of DR (Amat et al., 2005). The DR from which originates the 5-HT projection to the dorsal striatum does not receive reciprocal innervation from the striatum indicating no direct modulation by striatal feedback (Casanovas et al., 1999).

### Impulsivity and compulsivity

In contrast to the lack of effect of systemic or intracortical M100907 and Ro-60-0175 on perseverative responding, M100907 and Ro60-0175 administered locally in the dm-STR reduced perseverative responding caused by R-CPP. As systemic injections of these 5-HT_2_ agents had no effect on R-CPP-induced perseverative over-responding it is conceivable that the reduction of perseverative responding in one brain area such as dm-STR, is compensated by opposite effects in other brain regions. In fact, M100907 injected in the ventral tegmental area (VTA) further enhanced perseverative responding caused by blockade of NMDA receptors in the mPFC (Agnoli and Carli, 2012). These findings are in keeping with evidence that different 5-HT receptor subtypes have distinct roles in the modulation of perseverative responses, depending on the type of cognitive process engaged by the task and brain area. For example, 5-HT in the PFC is not essential for higher-order shifting of attentional set while it is critical for the flexible responding in a reversal learning task (Clarke et al., 2005). The 5-HT_2A_ and 5-HT_2C_ receptors exert functionally opposing action on perseverative responding in a spatial-reversal task (Boulougouris et al., 2008) while suppression of 5-HT_2C_ receptors in the orbitofrontal cortex but not in PFC decreases perseverative errors in a similar reversal-learning task (Boulougouris and Robbins, 2010).

It is worth noting that the ability of intra-STR M100907 and Ro60-0175 to remove impulsivity and compulsivity induced by blockade of mPFC NMDA receptors is remarkably similar to what found after systemic or intra dm-STR injections of a D_2_ receptor antagonist haloperidol (Baviera et al., 2008; Agnoli et al., 2013). It should be noted that blockade of NMDA receptors in the mPFC increases glutamate, GABA, and DA release in the dm-STR (see Table 2). As pointed above substantial neurochemical and behavioral evidence support the suggestion that 5-HT can influence DA's effects in the striatum, which may be of relevance for the observed analogy in the behavioral effects 5-HT_2A_ and 5-HT_2C_ receptor agents and D_2_ receptor antagonists. However, as M100907 and Ro60-0175 but not haloperidol had additional effects on accuracy deficit other likely non-D_2_, mechanisms in the dm-STR may also contribute. Local infusion of M100907 has been shown to decrease basal and MPTP-stimulated glutamate levels in the dorsal striatum and ameliorate behavioral impairment of MPTP-treated mice (Ansah et al., 2011).

Activation of 5-HT receptors in the striatum elicits predominantly inhibitory responses in the medium spiny (MS) projection neurons (el Mansari et al., 1994; el Mansari and Blier, 1997). 5-HT though 5-HT_2C_ excites striatal ACh interneurons, which in turn inhibit the glutamatergic input to MS projection neurons (Pakhotin and Bracci, 2007). Notably changes in firing activity of ACh interneurons encode behaviorally relevant information (Morris et al., 2004; Yamada et al., 2004). Activation of 5-HT_2C_ receptors strongly increases the firing of GABAergic interneurons in the striatum, which potently inhibit striatal output (Blomeley and Bracci, 2009). Thus, 5-HT_2_ receptor subtypes through a likely action on glutamate, ACh and GABA mechanisms in the dm-STR may integrate the glutamate cortico-striatal inputs critical for the different aspects of performance in the 5-CSRT task.

## Dopamine/NMDA receptors interaction and attention performance

### Dorsal striatal D_1_- and D_2_-like receptors

DA receptors are broadly expressed in the brain with a distribution largely matching the density of innervating DA fibers (Bentivoglio and Morelli, 2005). Among the DA receptors the D_1_ and D_2_ receptor subtypes display the widespread distribution and the highest expression levels. They are most prominent in the dorsal and ventral striatum, olfactory tubercle, and cortex (Bentivoglio and Morelli, 2005).

In the striatum the D_1_ and D_2_ receptors are segregated to the two MSN output populations of neurons forming direct striato-nigral and indirect striato-pallidal patways, respectively. However, both D_1_ and D_2_ receptors are expressed in a subset of MSN neurons in the striatum. Whether cooperative effects of D_1_ and D_2_ receptors observed in some studies (Perreault et al., 2011) arise from complex network interactions or from their co-localization in some MSN neurons is unclear. Striatal interneurons although proportionally small (5–10% of total neuronal population) exert powerful influence on striatal output. Five distinct GABA subtypes (distinguished by different neuropeptide expression, synthetic enzymes and calcium binding proteins) and one type of ACh interneurons are present. D_2_ and D_1_-like (D_5_) receptors are expressed in ACh interneurons while some GABA interneurons express D_1_-like (D_5_) receptors. D_2_ receptor is expressed also on presynaptic DA terminals of DA afferents as well as in glutamatergic cortical and thalamic afferents. D_1_ receptors have also been found in a small number of presynaptic glutamatergic terminals (Bentivoglio and Morelli, 2005).

Numerous studies have demonstrated that DA through pre- and post-synaptic D_1_ and D_2_ receptors modulate the probability of release at glutamate, GABA and ACh terminals, ionotropic glutamate, and GABA receptor function and trafficking, post-synaptic excitability and synaptic integration in striatal projecting neurons and interneurons as well as in cortical pyramidal cells and interneurons. DA bi-directionally modulates synaptic NMDA receptors through its D_1_- and D_2_-like receptors, but the responses of individual neurons across brain areas and the intracellular pathways recruited vary greatly. These studies (for a comprehensive review see Surmeier et al., 2010; Tritsch and Sabatini, 2012) reveal the complex nature and consequences of this modulation on neural networks implicated in motor, cognitive, and motivational processes (Di Chiara, 2005; Dunnett, 2005; Robbins, 2005; Berridge, 2007; Salamone and Correa, 2012).

## Performance in the 5-CSRT task

The effects of D_1_- and D_2_-like receptor antagonists SCH23390 and haloperidol injected in the dm-STR on accuracy and executive deficits induced by R-CPP injections in the mPFC are summarized in Table 4.

### Accuracy

In rats in which accuracy was impaired by intra-mPFC injections of R-CPP (50 ng/side) suppression of D_1_-like receptor activity in the dm-STR by an antagonist such as SCH23390 prevented accuracy deficit. It could be argued that in rats performing the 5-CSRT task at a very high level of efficiency (i.e., high accuracy) the activity at dorsal striatal D_1_-like receptors may be already at a maximum and any further activation such as that operated by intra-mPFC injections of R-CPP (Table 2) may have detrimental effects. On the other hand, concomitant infusion of SCH23390 and R-CPP in the dm-STR at individually ineffective doses had detrimental effects on accuracy (Agnoli and Carli, 2011). Thus, suppression of D_1_ receptor function in the dm-STR has positive or detrimental effects on accuracy depending on whether cortico-striatal neurotransmission is increased or decreased. Interestingly, another study also reported that systemic SCH23390 tend to improve accuracy of rats with excitotoxic lesion to mPFC (Passetti et al., 2003b).

These findings stand rather alone as in the majority of published studies the effects of D_1_-like agents was examined in normal rats performing the task under baseline conditions. The picture that emerges is that detrimental effects of SCH23390 on accuracy of rats performing the task under basal conditions depend on dose, brain area, and baseline level of accuracy (Granon et al., 2000; Pezze et al., 2007). In rats performing the task at relatively high level of accuracy (between 80 and 90%) SCH23390 injected in the mPFC or NAC impairs accuracy (Granon et al., 2000; Pezze et al., 2007) while the same dose (100 ng) injected in the dm-STR had no effect (Agnoli and Carli, 2011). Similarly the effect of SKF38393 a D_1_-like receptor agonist on accuracy is also baseline dependent; injected in the dm-STR or given systemically impairs accuracy of rats performing at high levels of efficiency (about 90%correct) whereas injected in the mPFC boosts accuracy but only in poorly performing rats (about 70% correct) (Granon et al., 2000). SKF38393 injected in the NAC boost accuracy of rats performing at less than 80% correct but only at the lowest dose tested (100 ng) while the same dose injected in the dorsolateral striatum had no effect. These discrepancies in the effects of D_1_ receptor agents on accuracy are far from surprising as it has been repeatedly shown that the effects of D_1_ receptor manipulation on task performance depend on the optimal levels of DA for the particular task (Sawaguchi and Goldman-Rakic, 1991; Arnsten, 1997; Zahrt et al., 1997; Granon et al., 2000; Pezze et al., 2003; Chudasama and Robbins, 2004; Robbins, 2005). This is reminiscent of the Yerkes-Dodson principle based on the inverted U-shaped function relating levels of arousal/activation with efficiency of behavioral performance (Robbins, 2005) but also to the inverted U-shaped function relating D_1_ receptor activation and NMDA-EPSC changes (Seamans and Yang, 2004; Trantham-Davidson et al., 2004; Tritsch and Sabatini, 2012). As for PFC an inverted U-shaped function may relate D_1_ receptor stimulation in the dm-STR to the efficiency of attentional functioning.

Contrasting with the findings above, suppression of D_2_-like receptor activity in the dm-STR by local injections of haloperidol has no effect in control rats and is unable to recover accuracy deficit caused by blockade of NMDA receptors in the mPFC (Agnoli et al., 2013). Similar lack of effects on R-CPP-induced accuracy deficit is observed after systemic haloperidol (Baviera et al., 2008). However, the doses used in these studies were effective in reversing other R-CPP-induced effects (see below). That D_2_-like receptors in the dorsal striatum are unlikely to be involved in governing accuracy is supported by data showing that their activation by quinpirole an agonist at these receptors has no effect either injected in the dorsomedial or dorsolateral striatum (Pezze et al., 2007; Agnoli et al., 2013). Doses of haloperidol and quinpirole higher than those reported in the studies by Baviera et al. (2008) and Agnoli et al. (2013) cannot be tested in the 5-CSRT task as rats stop responding or make mostly omissions.

Systemic haloperidol do not allows for the precise definition of the locus of D_2_ receptor suppression. However, after systemic administration haloperidol binds comparable proportion of D_2_ receptors in the striatum (caudate-putamen) and in the frontal cortex (Mukherjee et al., 2001) and the protein structure of the D_2_ receptors throughout the brain are similar and so is their *in vitro* affinity (Seeman and Ulpian, 1983). It is worth noting that a chemically different D_2_-like receptor antagonist such as l-sulpiride injected in the mPFC had no effect on accuracy (Granon et al., 2000). Although these findings may suggest that cortical D_2_-like receptors do not contribute to accuracy further studies are necessary to better delineate the role of PFC D_2_ receptors in attention. However, l-sulpiride given systemically or in the NAC impairs accuracy in control rats but prevents accuracy deficit in rats bearing excitotoxic lesions of the mPFC (Passetti et al., 2003b; Pezze et al., 2009). L-sulpiride does not discriminate between D_2_ and D_3_ receptor subtypes (Missale et al., 1998). The D_3_ receptors are present at very low levels in the mPFC and dorsal striatum but are particularly abundant in the NAC and limbic regions (Sokoloff et al., 1990; Bentivoglio and Morelli, 2005) and hence it could not be excluded the possibility that D_3_ receptors in the NAC may account for the effect of l-sulpiride. However, as systemic or intra-NAC nafadotride, a preferential D_3_ (compared to D_2_) receptor antagonist (Sautel et al., 1995) has no effect on accuracy (Besson et al., 2010) the precise contribution of D_3_ receptors for the control of accuracy has yet to be fully disclosed.

Intra-dm-STR injections of haloperidol or SCH23390 did not reduce the R-CPP-induced increase in omissions and correct response latencies. It is unlikely that the increased proportion of omissions was due to a change in motivation as the latency to collect the food, which is a more direct measure of motivation was not affected. This increase in omissions may indicate an inability to maintain voluntary control over sustained performance due to motor hyperactivity. However, haloperidol and SCH23390 did not reduce R-CPP-induced motor hyperactivity (Agnoli, 2011). SKF38393 speeded correct responses and decreased omissions when injected in the dm-STR (see Table 2 in Agnoli et al., 2013) similarly to what reported after intra-mPFC injection of this compound while systemic SKF38393 decrease correct response latencies (Passetti et al., 2003a). These finding are broadly consistent with a general performance scaling function of tonic DA activity (Cagniard et al., 2006) and with evidence from other reaction time tasks that striatal DA is implicated in decisional processes (Carli et al., 1985, 1989; Robbins and Brown, 1990; Brown and Robbins, 1991).

### Impulsivity and compulsivity

Dorsomedial striatal D_1_-like and D_2_-like receptors play an important role in the expression of impulsivity in the 5-CSRT task as both SCH23390 and haloperidol injected in the dm-STR dose-dependently reversed R-CPP-induced premature over-responding, a proxy of impulsivity. Blockade of these D_1_- and D_2_-like receptors in control conditions had no effect or decrease premature responses depending on the dose employed and the number of premature responses made by rats under the control condition (Agnoli and Carli, 2011; Agnoli et al., 2013) while their activation by SKF38393 and quinpirole, respectively increase premature responses (Agnoli et al., 2013). The finding that R-CPP-induced motor hyperactivity was not affected by SCH23390 and haloperidol (Agnoli, 2011) suggest that their ability to decrease R-CPP-induced impulsivity is not due to alteration in motor activity and helps dissociating impulsivity from changes in motor activity.

These findings question the prevailing hypothesis that impulsivity can be mostly attributed to the mesolimbic not the nigrostriatal DA system as d-amphetamine-induced impulsivity in the 5-CSRT task was abolished by ventral striatal but not dorsal striatal DA depletion (Cole and Robbins, 1989; Baunez and Robbins, 1999). In addition other studies had shown that D_1_- but not D_2_-like receptors in the NAC core contribute to impulsivity under basal conditions (Pezze et al., 2007) whereas D_2_-like receptors in the NAC appear to come into play only under perturbed conditions such as those induced by amphetamine or in rats made impulsive by excitotoxic lesion of the PFC (Cole and Robbins, 1987; Pattij et al., 2007; Pezze et al., 2009). In the case of high-impulsive rats D_2/3_ antagonist nafadotride alleviated or exacerbated impulsivity depending whether injected in the core or shell sub-region of the NAC, respectively (Besson et al., 2010). However, DA depletion in the dorsal striatum reversed impulsivity in the 5-CSRT task induced by lesions to the sub-thalamic nucleus (Baunez and Robbins, 1999) and D_2_ receptor availability in the dorsal striatum was associated with impulsiveness in methamphetamine-dependent subjects (Lee et al., 2009). Thus, the modulation of impulsivity by DA mechanisms in the dorsal striatum may be detected in particular conditions.

Haloperidol but not SCH23390 injected in the dm-STR reduce perseverative responding induced by blockade of NMDA in the mPFC. The effects of systemic and intra-dm-STR injected haloperidol are remarkably similar; both reduce perseverative and premature over-responding but not accuracy deficit (see, Tables 3, 4). These findings are in accord with a study showing that the “compulsive” stimulus bound perseveration of monkeys after frontal ablation is also alleviated by haloperidol (Ridley et al., 1993). However, other studies report that l-sulpiride either after systemic or intra-NAC injections had no effect in rats made compulsive by excitotoxic lesions of mPFC (Passetti et al., 2003b; Pezze et al., 2009). The causes for the lack of effect of this D_2_/D_3_ antagonist are not clear and may depend on various factors such as whether emitting a perseverative responses leads to behavioral consequence or not, brain area or else; for example it has been repeatedly shown that similar pharmacological manipulations increase perseverative responding when these lead to time-out but not when they are without consequences (Harrison et al., 1999; Robbins, 2002; Mirjana et al., 2004; Winstanley et al., 2004a; Murphy et al., 2005).

On the other hand, in normal rats performing the 5-CSRT task at baseline conditions activation of D_2_-like receptors in the dm-STR dose-dependently increase perseverative responding (Agnoli et al., 2013) similarly to what found after injections of similar doses of quinpirole in the NAC core but not after injections in the dorsolateral striatum (Pezze et al., 2007). That the perseverative responses in the 5-CSRT task may be modulated by nigrostriatal DA system is also suggested by paradoxical increase in these responses after dorsal striatal DA depletion (Baunez and Robbins, 1999) most likely due to the supersensivity of D_2_-receptors after 6-hydroxydopamine lesion (Ungerstedt, 1971). These findings are in agreement with other studies linking changes in D_2_ receptors function at various nodes of cortico-striatal circuit to flexible modification of behavior. Although it could not be assumed that perseverative errors in the 5-CSRT task and those made in other tasks such as for example in set-shifting, reversal learning or working memory represent the same psychological process, Floresco et al. (2006) report increased number of perseverative errors after blockade of D_2_-like receptors in the mPFC in a maze based set-shifting task while Goto and Grace (2005) report that PFC-dependent perseveration in a task requiring an egocentric response strategy depends on tonic DA release and D_2_-like receptor stimulation in the striatum. In addition, mice over-expressing D_2_ receptors in the striatum make more perseverative errors in a working memory task (Kellendonk et al., 2006). D_2_-receptor stimulation by quinpirole increases preseverative but not learning errors of rats performing a spatial reversal task (Boulougouris et al., 2009). The probability of perseverative responses of monkeys performing a three-choice reversal task is also related to D_2_-receptor availability in the dorsal striatum (Groman et al., 2011).

The lack of effects of D_1_-like receptor agents injected either systemically, in the mPFC, NAC or dm-STR on perseverative responding in the 5-CSRT task (Granon et al., 2000; Pezze et al., 2007; Agnoli and Carli, 2011; Barnes et al., 2012a; Agnoli et al., 2013) contrast with evidence that D_1_-like receptors in the mPFC or NAC control perseverative type errors in set-shifting and working memory tasks (Zahrt et al., 1997; Ragozzino, 2002; Haluk and Floresco, 2009). Thus, both DA receptor subtypes act in a cooperative manner to control a component of set-shifting such as ability to disengage from the previously effective but now inappropriate strategy whereas in the 5-CSRT task they appear to control separate cognitive processes such as those engaged by accuracy of visual discrimination and perseverative responding. However, the perseverative over-responding in the 5-CSRT task may result from a deficit in the selection and integration of an adequate response in a long sequence, leading to reward rather than the inability to flexibly adapt to the shifts between rules, strategies and sets. The organization of complex sequences of actions and the ordering of movements within a sequence implicate dorsal striatum with its DA afferents (Graybiel, 1998; Hikosaka et al., 1998; Bailey and Mair, 2006; Jin and Costa, 2010; Jin et al., 2014). Notably, the D_1_-nigrostriatal and D_2_-striatopallidal basal ganglia pathways show concomitant activity during action selection and initiation but behave differently during the execution of action sequences (Jin et al., 2014).

## Attention impairment and glutamate release in the mPFC

One of the characteristics of the microdialysis technique is the possibility to deliver drugs through the probe while collecting neurotransmitters generated and secreted by cells. In our microdialysis studies unilateral perfusion of 100 μM R-CPP through the probe in the mPFC for 1 h evoked a marked and reliable increase of glutamate, 5-HT and DA and a reduction of GABA therein (Tables 2, 3). However, the non-competitive NMDA receptor antagonists dizocilpine and ketamine increased cortical 5-HT efflux after bilateral but not unilateral infusion into the mPFC (Lopez-Gil et al., 2012). Although different administration techniques were used in behavioral (intraparenchimal injection) and microdialysis (perfusion through the probe) studies, the total amount of R-CPP delivered were similar (see Discussion in Calcagno et al., 2009). In addition, extracellular glutamate increased to a similar extent after R-CPP perfusion through the probe or intraparenchimal injection of the drug (50 ng/side) at the same dose as used in behavioral studies (see Figure S2 in Calcagno et al., 2009). This strengthens the link between microdialysis and behavioral data.

The proposal that excessive prefronto-cortical glutamate release plays a key role in cognitive deficit stems from the study by Moghaddam and Adams (1998) and is fuelled by a series of observation summarized in Table 3. While impulsivity and compulsivity do not appear to be associated with glutamate release in the PFC, Table 3 illustrate a tight association between the ability of several compounds to prevent R-CPP-induced attention deficits in the 5-CSRT task and the stimulation of glutamate release in the rat mPFC. The first evidence for this association was obtained with the selective 5-HT_2A_ receptors antagonist M100907. It was found that the same systemic doses of M100907 preventing attention deficit in the 5-CSRTT abolished the R-CPP-induced glutamate relase in the mPFC (Ceglia et al., 2004). However, another study failed to observe such interaction (Adams and Moghaddam, 2001). The perfusion of M100907 through the probe mimicked the effect of systemic injection in suppressing R-CPP- (Ceglia et al., 2004) and dizocilpine-induced rise of extracellular glutamate in the mPFC (Lopez-Gil et al., 2007). These findings indicate that cortical 5-HT_2A_ receptors may play a major role and that the stimulation of glutamate release may play a role in the attentional performance deficits caused by NMDA receptor blockade. Cortical 5-HT_1A_ and 5-HT_2A_ receptors co-localize in most pyramidal neurons of the mPFC (Santana et al., 2004) and exert opposite effect on their excitability (Araneda and Andrade, 1991; Ashby et al., 1994), head-twitches behavior (Darmani et al., 1990) and cortical dopamine release induced by D_2_ receptor blockade (Ichikawa et al., 2001). On this basis, it is expected that 5-HT_1A_ receptor stimulation ameliorate attention deficit induced by R-CPP (Carli et al., 2006a) by a mechanism similar to that of M100907. This was confirmed showing that intracortically perfused 8-OH-DPAT, a relatively selective 5-HT_1A_ receptors agonist, shared with M100907 the ability to prevent R-CPP-induced glutamate release in the mPFC (Calcagno et al., 2006). WAY100635 antagonized the effect of 8-OH-DPAT on glutamate release suggesting a selective involvement of 5-HT_1A_ receptors (Calcagno et al., 2006). These data were recently confirmed showing that dizocilpine-induced release of glutamate and 5-HT in the mPFC were suppressed by Bay × 3702, a 5-HT_1A_ receptor agonist (Lopez-Gil et al., 2009) and strengthen the suggestion that excessive glutamate in the mPFC is deleterious for attentional performance. Further support comes from studies showing that the 5-HT_2C_ receptor agonist Ro60-0175 mimicked M100907 in suppressing R-CPP-evoked glutamate release while the 5-HT_2C_ receptor antagonist SB242084 prevented the effect of M100907 on glutamate (Calcagno et al., 2009). This is not surprising in view of the well-recognized functional opposition between 5-HT_2A_ and 5-HT_2C_ receptors (Millan et al., 1998; Gobert and Millan, 1999) and suggests that 5-HT_2C_ receptors play a major role in controlling the effect of R-CPP on cortical glutamate release. Interestingly, R-CPP-induced rise of extracellular glutamate and 5-HT in the rat mPFC was prevented by M100907 and 5-HT depletion abolished these effects (Calcagno et al., 2009). Likewise, endogenous 5-HT is necessary for M100907 to inhibit motor activity induced by dizocilpine in mice (Martin et al., 1998). Although the effect of R-CPP on 5-HT is not related to its ability to impair attention or executive control (see Table 3), it could be argued that enhanced 5-HT tone on cortical 5-HT_1A_ may contribute to the ability of M100907 to counteract the effect of R-CPP on glutamate. However, failure of WAY100635 to prevent the effect of M100907 on R-CPP-induced glutamate release (Calcagno et al., 2009) rules this out. Thus, it is likely that M100907 suppresses glutamate release induced by R-CPP by enhancing the action of endogenous 5-HT on 5-HT_2C_ receptors. Taken together, these findings suggest that an imbalance in the control exerted by endogenous 5-HT on different receptor subtypes, rather than an action at a single receptor, determines the effect of NMDA antagonists on glutamate release and behavior.

The role of glutamate release in attention performance is further supported by data showing that the activation of pre-synaptic mGlu2/3 receptors, which suppress glutamate release, was sufficient to reduce R-CPP-induced accuracy deficits in the 5-CSRT task (Table 3). Similarly, the stimulation of mGlu2/3 receptors prevented the working memory impairment induced by PCP in the T-maze (Moghaddam and Adams, 1998).

Antipsychotic drugs show a complex pharmacology involving actions at different neurotransmitter receptors including agonist, antagonist, or partial agonist interactions with 5-HT_1A_, 5-HT_2A_, 5-HT_2C_ receptors (Arnt and Skarsfeldt, 1998), which may influence the effects of NMDA receptor antagonists on attention and cortical glutamate. In the 5CSRT task clozapine, olanzapine and low doses of sertindole prevented R-CPP-induced impairment of correct responses and impulsivity but had no effects on compulsivity (Baviera et al., 2008; Carli et al., 2011a,b) resembling the effect of M100907. These antipsychotics block with high affinity 5-HT_2A_ receptors (Arnt and Skarsfeldt, 1998), which likely played a major role in their effects on attention. Aripiprazole effect in the 5CSRT task resembled that of 8-OH-DPAT (see Table 3) suggesting an involvement of 5-HT_1A_ receptor stimulation (Carli et al., 2011b). Regardless of their precise mechanism of action, we found that clozapine, olanzapine, sertindole (low doses), and aripiprazole, which share the ability to counteract attention deficit induced by R-CPP, consistently suppressed R-CPP evoked glutamate release in the mPFC while 0.1 mg/kg haloperidol, which occupies most brain D_2_ receptors (Mukherjee et al., 2001) and 2.5 mg/kg sertindole, did not reverse attention deficits and had no effect of glutamate release (Table 3). In line with these findings, other studies showed that clozapine and olanzapine prevented dizocilpine-induced glutamate release (Lopez-Gil et al., 2007). Although 0.3 and higher doses of haloperidol reversed R-CPP and dizocilpine effects on glutamate (Lopez-Gil et al., 2009; Carli et al., 2011b), at 0.3 mg/kg rats stop responding or make mostly omissions, so their effects in the 5-CSRT task could not be reliably assessed. It should be emphasized that the same doses of drugs were used in our behavioral and microdialysis studies. This contributes to support the proposal that excessive glutamate release in the mPFC is deleterious for attention.

## Conclusions

In this review a special emphasis was given to distinct processes that govern the performance of rats in the 5-CSRT task. It is apparent that the input selection process of attention and executive control over impulsive and perseverative responding may be the results of integration of NMDA receptor function and the activity in 5-HT and DA receptor systems along the nodes of cortico-striatal circuitry.

Blockade of NMDA receptors in the mPFC induces a profound deficit in rat's performance in the 5-CSRT task characterized by impaired attention, increased impulsivity and perseverative responding and hyperactivation of cortico-striatal transmission. The reviewed studies show that these deficits are differentially responsive to pharmacological manipulations of 5-HT and DA receptor activity in the mPFC and dm-STR and that increased cortical glutamate release and cortico-striatal transmission is associated specifically with impaired attention but not with enhanced impulsivity and perseverative responding.

Direct comparison of the effects of various 5-HT_1A_, 5-HT_2A_, and 5-HT_2C_ agonists and antagonists most clearly implicate these 5-HT receptors in the mPFC in the preservation of input selection process of attention. Impulsivity in the 5-CSRT task, which has been definitely linked to changes in 5-HT function (Dalley and Roiser, 2012) is best controlled by suppression of 5-HT_2A_ or activation of 5-HT_2C_ receptors. In contrast, perseverative response deficit appear to be responsive to activation of 5-HT_1A_ receptor in the mPFC and the suppression of 5-HT_2A_ and activation of 5-HT_2C_ receptors in the dm-STR and VTA. In view of the well-recognized control of striatal and cortical DA function by 5-HT_1A_ and 5-HT_2_ receptors and the similar effects of a D_2_-like receptor antagonist such as haloperidol on perseverative response deficit, it is likely that this 5-HT receptors' control of perseverative responding may be the result of a functional interaction with D_2_-like receptor mechanisms. Manipulation of 5-HT receptors in another task putatively employed to evaluate similar processes confirms that, depending on the cortical area, 5-HT_2A_ and 5-HT_2C_ receptors exert functionally opposing action on perseverative responding (Boulougouris et al., 2008; Boulougouris and Robbins, 2010). Together, these studies highlight the complexity but also specificity of influences that 5-HT exert on prefrontal control of attention and executive functions depending on the receptor subtype, brain areas and specific processes engaged by the task.

The studies reviewed here also show a clear-cut dissociation in the roles played by dm-STR D_1_-like and D_2_-like receptors in the control of accuracy and perseverative responding. There is a definite relation between D_1_ receptor and attention but this relationship is not linear as it can be influenced by many factors such as the levels of baseline performance and optimal levels of DA for the performance of a particular task (Robbins, 2005). While accuracy is not responsive to D_2_-like receptor activity, the suppression of D_1_ receptor activity may improve or impair accuracy depending on the activity in the cortico-striatal transmission. The sensitivity of input selection process of attention to D1-like receptor manipulation in the dm-STR is in marked contrast to the lack of effect on processes underlying a form of behavioral flexibility such as that indexed by perseverative responses. Although there appears to be some overlap between D_1_-like and D_2_-like receptors in the modulation of certain domains of behavioral flexibility such as that involved in the ability to flexibly adapt to shift between rules, strategies, and sets (Floresco and Jentsch, 2011) the studies reviewed here clearly show that a different form of behavioral flexibility, which may result from the inability to select and integrate an adequate response in a long sequence leading to reward, is under control of D_2_-like but not D_1_-like receptor activity in the dm-STR. The two dorsal striatal DA receptor subtypes appear to act in a cooperative manner to control a different component of executive control such as impulsivity.

The suggestion emerging from this review is that the differential modulation of attention and executive functions by the 5-HT and DA systems highlights a degree of specificity for these “nonspecific” neurochemical pathways. These systems integrate the information conveyed by cortical pyramidal neurons at the level of functional modules, which are engaged selectively to optimize the operations necessary for the attentional and executive control over performance. The PFC controls the activity in these neurochemical pathways that in-turn they themselves modulate suggesting that this reciprocal control is essential for cognition.

The impairment in the 5-CSRT task performance by NMDA receptor antagonist administration in the mPFC may represent a model of attentional and executive dysfunction useful to explore the role of brain circuits and neurotransmitter systems in the cognitive symptoms of neuropsychiatric disorders.

### Conflict of interest statement

The authors declare that the research was conducted in the absence of any commercial or financial relationships that could be construed as a potential conflict of interest.

## References

[B1] Abdallah L. Bonasera S. J. Hopf F. W. O'Dell L. Giorgetti M. Jongsma M. (2009). Impact of serotonin 2C receptor null mutation on physiology and behavior associated with nigrostriatal dopamine pathway function. J. Neurosci. 29, 8156–8165 10.1523/JNEUROSCI.3905-08.2009 19553455 PMC3077993

[B2] Abekawa T. Ito K. Koyama T. (2006). Role of the simultaneous enhancement of NMDA and dopamine D1 receptor-mediated neurotransmission in the effects of clozapine on phencyclidine-induced acute increases in glutamate levels in the rat medial prefrontal cortex. Naunyn Schmiedebergs Arch. Pharmacol. 374, 177–193 10.1007/s00210-006-0115-9 17103144

[B3] Abi-Saab W. M. Bubser M. Roth R. H. Deutch A. Y. (1999). 5-HT2 receptor regulation of extracellular GABA levels in the prefrontal cortex. Neuropsychopharmacology 20, 92–96 10.1016/S0893-133X(98)00046-3 9885788

[B4] Adams B. W. Moghaddam B. (2001). Effect of clozapine, haloperidol, or M100907 on phencyclidine-activated glutamate efflux in the prefrontal cortex. Biol. Psychiatry 50, 750–757 10.1016/S0006-3223(01)01195-7 11720693

[B5] Aghajanian G. K. Marek G. J. (1997). Serotonin induces excitatory postsynaptic potentials in apical dendrites of neocortical pyramidal cells. Neuropharmacology 36, 589–599 10.1016/S0028-3908(97)00051-8 9225284

[B6] Aghajanian G. K. Marek G. J. (1999). Serotonin, via 5-HT2A receptors, increases EPSCs in layer V pyramidal cells of prefrontal cortex by an asynchronous mode of glutamate release. Brain Res. 825, 161–171 10.1016/S0006-8993(99)01224-X 10216183

[B7] Aghajanian G. K. Marek G. J. (2000). Serotonin model of schizophrenia: emerging role of glutamate mechanisms. Brain Res. Brain Res. Rev. 31, 302–312 10.1016/S0165-0173(99)00046-6 10719157

[B8] Agnoli L. (2011). Modulation of Cortical Cognitive Functions by Dopamine and Serotonin Receptors in the Dorsal Striatum. Ph.D. thesis, In Life Science, Open University, Milton Keynes, 300

[B9] Agnoli L. Carli M. (2011). Synergistic interaction of dopamine D(1) and glutamate N-methyl-d-aspartate receptors in the rat dorsal striatum controls attention. Neuroscience 185, 39–49 10.1016/j.neuroscience.2011.04.044 21536111

[B10] Agnoli L. Carli M. (2012). Dorsal-striatal 5-HT(2)A and 5-HT(2)C receptors control impulsivity and perseverative responding in the 5-choice serial reaction time task. Psychopharmacology (Berl). 219, 633–645 10.1007/s00213-011-2581-0 22113450

[B11] Agnoli L. Mainolfi P. Invernizzi R. W. Carli M. (2013). Dopamine D1-like and D2-like receptors in the dorsal striatum control different aspects of attentional performance in the five-choice serial reaction time task under a condition of increased activity of corticostriatal inputs. Neuropsychopharmacology 38, 701–714 10.1038/npp.2012.236 23232445 PMC3671986

[B12] Ago Y. Sakaue M. Baba A. Matsuda T. (2002). Selective reduction by isolation rearing of 5-HT1A receptor-mediated dopamine release *in vivo* in the frontal cortex of mice. J. Neurochem. 83, 353–359 10.1046/j.1471-4159.2002.01128.x 12423245

[B13] Amat J. Baratta M. V. Paul E. Bland S. T. Watkins L. R. Maier S. F. (2005). Medial prefrontal cortex determines how stressor controllability affects behavior and dorsal raphe nucleus. Nat. Neurosci. 8, 365–371 10.1038/nn1399 15696163 PMC13675003

[B14] Amitai N. Markou A. (2010). Disruption of performance in the five-choice serial reaction time task induced by administration of N-methyl-D-aspartate receptor antagonists: relevance to cognitive dysfunction in schizophrenia. Biol. Psychiatry 68, 5–16 10.1016/j.biopsych.2010.03.004 20488434 PMC2900523

[B15] Amitai N. Semenova S. Markou A. (2007). Cognitive-disruptive effects of the psychotomimetic phencyclidine and attenuation by atypical antipsychotic medications in rats. Psychopharmacology (Berl). 193, 521–537 10.1007/s00213-007-0808-x 17497138

[B16] Ansah T. A. Ferguson M. C. Nayyar T. (2011). The 5-HT(2A) receptor antagonist M100907 produces antiparkinsonian effects and decreases striatal glutamate. Front. Syst. Neurosci. 5:48 10.3389/fnsys.2011.00048 21716656 PMC3117200

[B17] Araneda R. Andrade R. (1991). 5-Hydroxytryptamine2 and 5-hydroxytryptamine 1A receptors mediate opposing responses on membrane excitability in rat association cortex. Neuroscience 40, 399–412 10.1016/0306-4522(91)90128-B 1851255

[B18] Arborelius L. Nomikos G. G. Hacksell U. Svensson T. H. (1993). (R)-8-OH-DPAT preferentially increases dopamine release in rat medial prefrontal cortex. Acta Physiol. Scand. 148, 465–466 10.1111/j.1748-1716.1993.tb09584.x 8213201

[B19] Arnsten A. F. (1997). Catecholamine regulation of the prefrontal cortex. J. Psychopharmacol. 11, 151–162 10.1177/026988119701100208 9208378

[B20] Arnsten A. F. Wang M. J. Paspalas C. D. (2012). Neuromodulation of thought: flexibilities and vulnerabilities in prefrontal cortical network synapses. Neuron 76, 223–239 10.1016/j.neuron.2012.08.038 23040817 PMC3488343

[B21] Arnt J. Skarsfeldt T. (1998). Do novel antipsychotics have similar pharmacological characteristics? A review of the evidence. Neuropsychopharmacology 18, 63–101 10.1016/S0893-133X(97)00112-7 9430133

[B22] Ashby C. R. Jr. Edwards E. Wang R. Y. (1994). Electrophysiological evidence for a functional interaction between 5-HT1A and 5-HT2A receptors in the rat medial prefrontal cortex: an iontophoretic study. Synapse 17, 173–181 10.1002/syn.890170306 7974200

[B23] Aston-Jones G. Rajkowski J. Cohen J. (2000). Locus coeruleus and regulation of behavioral flexibility and attention. Prog. Brain Res. 126, 165–182 10.1016/S0079-6123(00)26013-5 11105646

[B24] Auclair A. L. Besnard J. Newman-Tancredi A. Depoortere R. (2009). The five choice serial reaction time task: comparison between Sprague-Dawley and Long-Evans rats on acquisition of task, and sensitivity to phencyclidine. Pharmacol. Biochem. Behav. 92, 363–369 10.1016/j.pbb.2009.01.005 19353758

[B25] Azmitia E. C. Segal M. (1978). An autoradiographic analysis of the differential ascending projections of the dorsal and median raphe nuclei in the rat. J. Comp. Neurol. 179, 641–667 10.1002/cne.901790311 565370

[B26] Bailey K. R. Mair R. G. (2006). The role of striatum in initiation and execution of learned action sequences in rats. J. Neurosci. 26, 1016–1025 10.1523/JNEUROSCI.3883-05.2006 16421321 PMC6675371

[B27] Balla A. Nattini M. E. Sershen H. Lajtha A. Dunlop D. S. Javitt D. C. (2009). GABAB/NMDA receptor interaction in the regulation of extracellular dopamine levels in rodent prefrontal cortex and striatum. Neuropharmacology 56, 915–921 10.1016/j.neuropharm.2009.01.021 19371582 PMC4681299

[B28] Barnes N. M. Sharp T. (1999). A review of central 5-HT receptors and their function. Neuropharmacology 38, 1083–1152 10.1016/S0028-3908(99)00010-6 10462127

[B29] Barnes S. A. Young J. W. Neill J. C. (2012a). D receptor activation improves vigilance in rats as measured by the 5-choice continuous performance test. Psychopharmacology (Berl). 220, 129–141 10.1007/s00213-011-2460-8 21901319 PMC5870138

[B30] Barnes S. A. Young J. W. Neill J. C. (2012b). Rats tested after a washout period from sub-chronic PCP administration exhibited impaired performance in the 5-choice continuous performance test (5C-CPT) when the attentional load was increased. Neuropharmacology 62, 1432–1441 10.1016/j.neuropharm.2011.04.024 21569782 PMC5870141

[B31] Baunez C. Robbins T. W. (1999). Effects of dopamine depletion of the dorsal striatum and further interaction with subthalamic nucleus lesions in an attentional task in the rat. Neuroscience 92, 1343–1356 10.1016/S0306-4522(99)00065-2 10426489

[B32] Baviera M. Invernizzi R. W. Carli M. (2008). Haloperidol and clozapine have dissociable effects in a model of attentional performance deficits induced by blockade of NMDA receptors in the mPFC. Psychopharmacology (Berl). 196, 269–280 10.1007/s00213-007-0959-9 17940750

[B33] Bentivoglio M. Morelli M. (2005). The organization and circuits of mesencephalic dopaminergic neurons and the distribution of dopamine receptors in the brain, in Dopamine, eds Dunnett S. B. Bentivoglio M. Bjorklund A. Hokfelt T. (Amsterdam: Elsevier), 1–107

[B34] Berridge K. C. (2007). The debate over dopamine's role in reward: the case for incentive salience. Psychopharmacology (Berl). 191, 391–431 10.1007/s00213-006-0578-x 17072591

[B35] Besson M. Belin D. McNamara R. Theobald D. E. Castel A. Beckett V. L. (2010). Dissociable control of impulsivity in rats by dopamine d2/3 receptors in the core and shell subregions of the nucleus accumbens. Neuropsychopharmacology 35, 560–569 10.1038/npp.2009.162 19847161 PMC3055378

[B36] Bishop C. Walker P. D. (2003). Combined intrastriatal dopamine D1 and serotonin 5-HT2 receptor stimulation reveals a mechanism for hyperlocomotion in 6-hydroxydopamine-lesioned rats. Neuroscience 121, 649–657 10.1016/S0306-4522(03)00516-5 14568025

[B37] Blomeley C. Bracci E. (2005). Excitatory effects of serotonin on rat striatal cholinergic interneurones. J. Physiol. 569, 715–721 10.1113/jphysiol.2005.098269 16269435 PMC1464279

[B38] Blomeley C. P. Bracci E. (2009). Serotonin excites fast-spiking interneurons in the striatum. Eur. J. Neurosci. 29, 1604–1614 10.1111/j.1460-9568.2009.06725.x 19419423 PMC2695856

[B39] Blue M. E. Yagaloff K. A. Mamounas L. A. Hartig P. R. Molliver M. E. (1988). Correspondence between 5-HT2 receptors and serotonergic axons in rat neocortex. Brain Res. 453, 315–328 10.1016/0006-8993(88)90172-2 3401769

[B40] Bonanno G. Fassio A. Schmid G. Severi P. Sala R. Raiteri M. (1997). Pharmacologically distinct GABAB receptors that mediate inhibition of GABA and glutamate release in human neocortex. Br. J. Pharmacol. 120, 60–64 10.1038/sj.bjp.0700852 9117099 PMC1564334

[B41] Boulougouris V. Castane A. Robbins T. W. (2009). Dopamine D2/D3 receptor agonist quinpirole impairs spatial reversal learning in rats: investigation of D3 receptor involvement in persistent behavior. Psychopharmacology (Berl). 202, 611–620 10.1007/s00213-008-1341-2 18836703

[B42] Boulougouris V. Glennon J. C. Robbins T. W. (2008). Dissociable effects of selective 5-HT2A and 5-HT2C receptor antagonists on serial spatial reversal learning in rats. Neuropsychopharmacology 33, 2007–2019 10.1038/sj.npp.1301584 17957219

[B43] Boulougouris V. Robbins T. W. (2010). Enhancement of spatial reversal learning by 5-HT2C receptor antagonism is neuroanatomically specific. J. Neurosci. 30, 930–938 10.1523/JNEUROSCI.4312-09.2010 20089901 PMC6633094

[B44] Brown V. J. Robbins T. W. (1991). Simple and choice reaction time performance following unilateral striatal dopamine depletion in the rat. Impaired motor readiness but preserved response preparation. Brain 114(pt 1B), 513–525 10.1093/brain/114.1.513 2004254

[B45] Buschman T. J. Miller E. K. (2007). Top-down versus bottom-up control of attention in the prefrontal and posterior parietal cortices. Science 315, 1860–1862 10.1126/science.1138071 17395832

[B46] Cagniard B. Beeler J. A. Britt J. P. McGehee D. S. Marinelli M. Zhuang X. (2006). Dopamine scales performance in the absence of new learning. Neuron 51, 541–547 10.1016/j.neuron.2006.07.026 16950153

[B47] Cai X. Gu Z. Zhong P. Ren Y. Yan Z. (2002). Serotonin 5-HT1A receptors regulate AMPA receptor channels through inhibiting Ca2+/calmodulin-dependent kinase II in prefrontal cortical pyramidal neurons. J. Biol. Chem. 277, 36553–36562 10.1074/jbc.M203752200 12149253

[B48] Calcagno E. Carli M. Baviera M. Invernizzi R. W. (2009). Endogenous serotonin and serotonin2C receptors are involved in the ability of M100907 to suppress cortical glutamate release induced by NMDA receptor blockade. J. Neurochem. 108, 521–532 10.1111/j.1471-4159.2008.05789.x 19046357

[B49] Calcagno E. Carli M. Invernizzi R. W. (2006). The 5-HT(1A) receptor agonist 8-OH-DPAT prevents prefrontocortical glutamate and serotonin release in response to blockade of cortical NMDA receptors. J. Neurochem. 96, 853–860 10.1111/j.1471-4159.2005.03600.x 16405507

[B50] Carli M. (2006b). Serotonergic Modulation of Attentional Processes in the Rat Prefrontal Cortex. PhD thesis, In Life Science, Open University. Milton Keynes, 290

[B51] Carli M. Baviera M. Invernizzi R. W. Balducci C. (2006a). Dissociable contribution of 5-HT1A and 5-HT2A receptors in the medial prefrontal cortex to different aspects of executive control such as impulsivity and compulsive perseveration in rats. Neuropsychopharmacology 31, 757–767 10.1038/sj.npp.1300893 16192987

[B52] Carli M. Bonalumi P. Samanin R. (1998). Stimulation of 5-HT1A receptors in the dorsal raphe reverses the impairment of spatial learning caused by intrahippocampal scopolamine in rats. Eur. J. Neurosci. 10, 221–230 10.1046/j.1460-9568.1998.00034.x 9753130

[B53] Carli M. Calcagno E. Mainini E. Arnt J. Invernizzi R. W. (2011a). Sertindole restores attentional performance and suppresses glutamate release induced by the NMDA receptor antagonist CPP. Psychopharmacology (Berl). 214, 625–637 10.1007/s00213-010-2066-6 21049266

[B54] Carli M. Calcagno E. Mainolfi P. Mainini E. Invernizzi R. W. (2011b). Effects of aripiprazole, olanzapine, and haloperidol in a model of cognitive deficit of schizophrenia in rats: relationship with glutamate release in the medial prefrontal cortex. Psychopharmacology (Berl). 214, 639–652 10.1007/s00213-010-2065-7 21052982

[B55] Carli M. Evenden J. L. Robbins T. W. (1985). Depletion of unilateral striatal dopamine impairs initiation of contralateral actions and not sensory attention. Nature 313, 679–682 10.1038/313679a0 3974701

[B56] Carli M. Jones G. H. Robbins T. W. (1989). Effects of unilateral dorsal and ventral striatal dopamine depletion on visual neglect in the rat: a neural and behavioural analysis. Neuroscience 29, 309–327 10.1016/0306-4522(89)90059-6 2498760

[B57] Carli M. Robbins T. W. Evenden J. L. Everitt B. J. (1983). Effects of lesions to ascending noradrenergic neurones on performance of a 5-choice serial reaction task in rats; implications for theories of dorsal noradrenergic bundle function based on selective attention and arousal. Behav. Brain Res. 9, 361–380 10.1016/0166-4328(83)90138-9 6639741

[B58] Carli M. Samanin R. (2000). The 5-HT(1A) receptor agonist 8-OH-DPAT reduces rats' accuracy of attentional performance and enhances impulsive responding in a five-choice serial reaction time task: role of presynaptic 5-HT(1A) receptors. Psychopharmacology (Berl). 149, 259–268 10.1007/s002139900368 10823407

[B59] Casanovas J. M. Hervas I. Artigas F. (1999). Postsynaptic 5-HT1A receptors control 5-HT release in the rat medial prefrontal cortex. Neuroreport 10, 1441–1445 10.1097/00001756-199905140-00010 10380960

[B60] Castner S. A. Williams G. V. (2007). Tuning the engine of cognition: a focus on NMDA/D1 receptor interactions in prefrontal cortex. Brain Cogn. 63, 94–122 10.1016/j.bandc.2006.11.002 17204357

[B61] Ceglia I. Carli M. Baviera M. Renoldi G. Calcagno E. Invernizzi R. W. (2004). The 5-HT receptor antagonist M100,907 prevents extracellular glutamate rising in response to NMDA receptor blockade in the mPFC. J. Neurochem. 91, 189–199 10.1111/j.1471-4159.2004.02704.x 15379899

[B62] Celada P. Puig M. V. Artigas F. (2013). Serotonin modulation of cortical neurons and networks. Front. Integr. Neurosci. 7:25 10.3389/fnint.2013.00025 23626526 PMC3630391

[B63] Celada P. Puig M. V. Casanovas J. M. Guillazo G. Artigas F. (2001). Control of dorsal raphe serotonergic neurons by the medial prefrontal cortex: involvement of serotonin-1A, GABA(A), and glutamate receptors. J. Neurosci. 21, 9917–9929 11739599 10.1523/JNEUROSCI.21-24-09917.2001 PMC6763042

[B64] Christakou A. Robbins T. W. Everitt B. J. (2001). Functional disconnection of a prefrontal cortical-dorsal striatal system disrupts choice reaction time performance: implications for attentional function. Behav. Neurosci. 115, 812–825 10.1037/0735-7044.115.4.812 11508720

[B65] Chudasama Y. Baunez C. Robbins T. W. (2003a). Functional disconnection of the medial prefrontal cortex and subthalamic nucleus in attentional performance: evidence for corticosubthalamic interaction. J. Neurosci. 23, 5477–5485 12843247 10.1523/JNEUROSCI.23-13-05477.2003 PMC6741240

[B66] Chudasama Y. Muir J. L. (2001). Visual attention in the rat: a role for the prelimbic cortex and thalamic nuclei? Behav. Neurosci. 115, 417–428 10.1037/0735-7044.115.2.417 11345966

[B67] Chudasama Y. Nathwani F. Robbins T. W. (2005). D-Amphetamine remediates attentional performance in rats with dorsal prefrontal lesions. Behav. Brain Res. 158, 97–107 10.1016/j.bbr.2004.08.011 15680198

[B68] Chudasama Y. Passetti F. Rhodes S. E. Lopian D. Desai A. Robbins T. W. (2003b). Dissociable aspects of performance on the 5-choice serial reaction time task following lesions of the dorsal anterior cingulate, infralimbic and orbitofrontal cortex in the rat: differential effects on selectivity, impulsivity and compulsivity. Behav. Brain Res. 146, 105–119 10.1016/j.bbr.2003.09.020 14643464

[B69] Chudasama Y. Robbins T. W. (2004). Dopaminergic modulation of visual attention and working memory in the rodent prefrontal cortex. Neuropsychopharmacology 29, 1628–1636 10.1038/sj.npp.1300490 15138446

[B70] Clarke H. F. Walker S. C. Crofts H. S. Dalley J. W. Robbins T. W. Roberts A. C. (2005). Prefrontal serotonin depletion affects reversal learning but not attentional set shifting. J. Neurosci. 25, 532–538 10.1523/JNEUROSCI.3690-04.2005 15647499 PMC6725478

[B71] Clemett D. A. Punhani T. Duxon M. S. Blackburn T. P. Fone K. C. (2000). Immunohistochemical localisation of the 5-HT2C receptor protein in the rat CNS. Neuropharmacology 39, 123–132 10.1016/S0028-3908(99)00086-6 10665825

[B72] Cole B. J. Robbins T. W. (1987). Amphetamine impairs the discriminative performance of rats with dorsal noradrenergic bundle lesions on a 5-choice serial reaction time task: new evidence for central dopaminergic-noradrenergic interactions. Psychopharmacology (Berl). 91, 458–466 10.1007/BF00216011 3108926

[B73] Cole B. J. Robbins T. W. (1989). Effects of 6-hydroxydopamine lesions of the nucleus accumbens septi on performance of a 5-choice serial reaction time task in rats: implications for theories of selective attention and arousal. Behav. Brain Res. 33, 165–179 10.1016/S0166-4328(89)80048-8 2504222

[B74] Compte A. Brunel N. Goldman-Rakic P. S. Wang X. J. (2000). Synaptic mechanisms and network dynamics underlying spatial working memory in a cortical network model. Cereb. Cortex 10, 910–923 10.1093/cercor/10.9.910 10982751

[B75] Conti F. Minelli A. DeBiasi S. Melone M. (1997). Neuronal and glial localization of NMDA receptors in the cerebral cortex. Mol. Neurobiol. 14, 1–18 10.1007/BF02740618 9170098

[B76] Corbett R. Zhou L. Sorensen S. M. Mondadori C. (1999). Animal models of negative symptoms: M100907 antagonizes PCP-induced immobility in a forced swim test in mice. Neuropsychopharmacology 21, S211–S218 10.1016/S0893-133X(99)00128-1

[B77] Czyrak A. Czepiel K. Mackowiak M. Chocyk A. Wedzony K. (2003). Serotonin 5-HT1A receptors might control the output of cortical glutamatergic neurons in rat cingulate cortex. Brain Res. 989, 42–51 10.1016/S0006-8993(03)03352-3 14519510

[B78] Dalley J. W. Everitt B. J. Robbins T. W. (2011). Impulsivity, compulsivity, and top-down cognitive control. Neuron 69, 680–694 10.1016/j.neuron.2011.01.020 21338879

[B79] Dalley J. W. Roiser J. P. (2012). Dopamine, serotonin and impulsivity. Neuroscience 215, 42–58 10.1016/j.neuroscience.2012.03.065 22542672

[B80] Dalley J. W. Theobald D. E. Eagle D. M. Passetti F. Robbins T. W. (2002). Deficits in impulse control associated with tonically-elevated serotonergic function in rat prefrontal cortex. Neuropsychopharmacology 26, 716–728 10.1016/S0893-133X(01)00412-2 12007742

[B81] Darmani N. A. Martin B. R. Pandey U. Glennon R. A. (1990). Do functional relationships exist between 5-HT1A and 5-HT2 receptors? Pharmacol. Biochem. Behav. 36, 901–906 10.1016/0091-3057(90)90098-3 2145593

[B82] David H. N. Ansseau M. Abraini J. H. (2005). Dopamine-glutamate reciprocal modulation of release and motor responses in the rat caudate-putamen and nucleus accumbens of “intact” animals. Brain Res. Brain Res. Rev. 50, 336–360 10.1016/j.brainresrev.2005.09.002 16278019

[B83] de Bartolomeis A. Buonaguro E. F. Iasevoli F. (2013). Serotonin-glutamate and serotonin-dopamine reciprocal interactions as putative molecular targets for novel antipsychotic treatments: from receptor heterodimers to postsynaptic scaffolding and effector proteins. Psychopharmacology (Berl). 225, 1–19 10.1007/s00213-012-2921-8 23179966

[B84] DeFelipe J. Arellano J. I. Gomez A. Azmitia E. C. Munoz A. (2001). Pyramidal cell axons show a local specialization for GABA and 5-HT inputs in monkey and human cerebral cortex. J. Comp. Neurol. 433, 148–155 10.1002/cne.1132 11283956

[B85] Del Arco A. Mora F. (1999). Effects of endogenous glutamate on extracellular concentrations of GABA, dopamine, and dopamine metabolites in the prefrontal cortex of the freely moving rat: involvement of NMDA and AMPA/KA receptors. Neurochem. Res. 24, 1027–1035 10.1023/A:1021056826829 10478942

[B86] Di Chiara G. (2005). Dopamine, motivation and reward, in Dopamine, eds Dunnett S. B. Bentivoglio M. Bjorklund A. Hokfelt T. (Amsterdam: Elsevier), 303–394

[B87] Dijk S. N. Francis P. T. Stratmann G. C. Bowen D. M. (1995). NMDA-induced glutamate and aspartate release from rat cortical pyramidal neurones: evidence for modulation by a 5-HT1A antagonist. Br. J. Pharmacol. 115, 1169–1174 10.1111/j.1476-5381.1995.tb15020.x 7582540 PMC1908786

[B88] Di Matteo V. Pierucci M. Esposito E. Crescimanno G. Benigno A. Di Giovanni G. (2008). Serotonin modulation of the basal ganglia circuitry: therapeutic implication for Parkinson's disease and other motor disorders. Prog. Brain Res. 172, 423–463 10.1016/S0079-6123(08)00921-7 18772045

[B89] Driesen N. R. McCarthy G. Bhagwagar Z. Bloch M. Calhoun V. D'Souza D. C. (2013a). Relationship of resting brain hyperconnectivity and schizophrenia-like symptoms produced by the NMDA receptor antagonist ketamine in humans. Mol. Psychiatry 18, 1199–1204 10.1038/mp.2012.194 23337947 PMC3646075

[B90] Driesen N. R. McCarthy G. Bhagwagar Z. Bloch M. H. Calhoun V. D. D'Souza D. C. (2013b). The impact of NMDA receptor blockade on human working memory-related prefrontal function and connectivity. Neuropsychopharmacology 38, 2613–2622 10.1038/npp.2013.170 23856634 PMC3828532

[B91] Dunnett S. B. (2005). Motor functions of the nigrostriatal dopamine system: studies of lesions and behaviour, in Dopamine, Dunnett S. B. Bentivoglio M. Bjorklund A. Hokfelt T. (Amsterdam: Elsevier), 237–302

[B92] Eberle-Wang K. Mikeladze Z. Uryu K. Chesselet M. F. (1997). Pattern of expression of the serotonin2C receptor messenger RNA in the basal ganglia of adult rats. J. Comp. Neurol. 384, 233–247 10.1002/(SICI)1096-9861(19970728)384:2<233::AID-CNE5>3.0.CO;2-2 9215720

[B93] Edagawa Y. Saito H. Abe K. (1998). Serotonin inhibits the induction of long-term potentiation in rat primary visual cortex. Prog. Neuropsychopharmacol. Biol. Psychiatry 22, 983–997 10.1016/S0278-5846(98)00055-4 9789882

[B94] el Mansari M. Blier P. (1997). *In vivo* electrophysiological characterization of 5-HT receptors in the guinea pig head of caudate nucleus and orbitofrontal cortex. Neuropharmacology 36, 577–588 10.1016/S0028-3908(97)00035-X 9225283

[B95] el Mansari M. Radja F. Ferron A. Reader T. A. Molina-Holgado E. Descarries L. (1994). Hypersensitivity to serotonin and its agonists in serotonin-hyperinnervated neostriatum after neonatal dopamine denervation. Eur. J. Pharmacol. 261, 171–178 10.1016/0014-2999(94)90316-6 8001641

[B96] Evenden J. L. (1999). Varieties of impulsivity. Psychopharmacology (Berl). 146, 348–361 10.1007/PL00005481 10550486

[B97] Feenstra M. G. Botterblom M. H. van Uum J. F. (2002). Behavioral arousal and increased dopamine efflux after blockade of NMDA-receptors in the prefrontal cortex are dependent on activation of glutamatergic neurotransmission. Neuropharmacology 42, 752–763 10.1016/S0028-3908(02)00029-1 12015201

[B98] Feng J. Cai X. Zhao J. Yan Z. (2001). Serotonin receptors modulate GABA(A) receptor channels through activation of anchored protein kinase C in prefrontal cortical neurons. J. Neurosci. 21, 6502–6511 11517239 10.1523/JNEUROSCI.21-17-06502.2001 PMC6763081

[B99] Fletcher P. J. Rizos Z. Noble K. Higgins G. A. (2011). Impulsive action induced by amphetamine, cocaine and MK801 is reduced by 5-HT(2C) receptor stimulation and 5-HT(2A) receptor blockade. Neuropharmacology 61, 468–477 10.1016/j.neuropharm.2011.02.025 21402085

[B100] Fletcher P. J. Tampakeras M. Sinyard J. Higgins G. A. (2007). Opposing effects of 5-HT(2A) and 5-HT(2C) receptor antagonists in the rat and mouse on premature responding in the five-choice serial reaction time test. Psychopharmacology (Berl). 195, 223–234 10.1007/s00213-007-0891-z 17673981

[B101] Floresco S. B. Jentsch J. D. (2011). Pharmacological enhancement of memory and executive functioning in laboratory animals. Neuropsychopharmacology 36, 227–250 10.1038/npp.2010.158 20844477 PMC3055518

[B102] Floresco S. B. Magyar O. Ghods-Sharifi S. Vexelman C. Tse M. T. (2006). Multiple dopamine receptor subtypes in the medial prefrontal cortex of the rat regulate set-shifting. Neuropsychopharmacology 31, 297–309 10.1038/sj.npp.1300825 16012531

[B103] Funahashi S. Bruce C. J. Goldman-Rakic P. S. (1989). Mnemonic coding of visual space in the monkey's dorsolateral prefrontal cortex. J. Neurophysiol. 61, 331–349 2918358 10.1152/jn.1989.61.2.331

[B104] Fuster J. M. (2009). Cortex and memory: emergence of a new paradigm. J. Cogn. Neurosci. 21, 2047–2072 10.1162/jocn.2009.21280 19485699

[B105] Gewirtz J. C. Marek G. J. (2000). Behavioral evidence for interactions between a hallucinogenic drug and group II metabotropic glutamate receptors. Neuropsychopharmacology 23, 569–576 10.1016/S0893-133X(00)00136-6 11027922

[B106] Gilmour G. Arguello A. Bari A. Brown V. J. Carter C. Floresco S. B. (2013). Measuring the construct of executive control in schizophrenia: defining and validating translational animal paradigms for discovery research. Neurosci. Biobehav. Rev. 37, 2125–2140 10.1016/j.neubiorev.2012.04.006 22548905

[B107] Gleason S. D. Shannon H. E. (1997). Blockade of phencyclidine-induced hyperlocomotion by olanzapine, clozapine and serotonin receptor subtype selective antagonists in mice. Psychopharmacology (Berl). 129, 79–84 10.1007/s002130050165 9122367

[B108] Gobert A. Millan M. J. (1999). Serotonin (5-HT)2A receptor activation enhances dialysate levels of dopamine and noradrenaline, but not 5-HT, in the frontal cortex of freely-moving rats. Neuropharmacology 38, 315–317 10.1016/S0028-3908(98)00188-9 10218874

[B109] Gold J. M. Fuller R. L. Robinson B. M. Braun E. L. Luck S. J. (2007). Impaired top-down control of visual search in schizophrenia. Schizophr. Res. 94, 148–155 10.1016/j.schres.2007.04.023 17544632 PMC1978542

[B110] Gonzalez-Maeso J. Ang R. L. Yuen T. Chan P. Weisstaub N. V. Lopez-Gimenez J. F. (2008). Identification of a serotonin/glutamate receptor complex implicated in psychosis. Nature 452, 93–97 10.1038/nature06612 18297054 PMC2743172

[B111] Goto Y. Grace A. A. (2005). Dopaminergic modulation of limbic and cortical drive of nucleus accumbens in goal-directed behavior. Nat. Neurosci. 8, 805–812 10.1038/nn1471 15908948

[B112] Granon S. Passetti F. Thomas K. L. Dalley J. W. Everitt B. J. Robbins T. W. (2000). Enhanced and impaired attentional performance after infusion of D1 dopaminergic receptor agents into rat prefrontal cortex. J. Neurosci. 20, 1208–1215 10648725 10.1523/JNEUROSCI.20-03-01208.2000 PMC6774157

[B113] Graybiel A. M. (1998). The basal ganglia and chunking of action repertoires. Neurobiol. Learn. Mem. 70, 119–136 10.1006/nlme.1998.3843 9753592

[B114] Green M. F. Kern R. S. Heaton R. K. (2004). Longitudinal studies of cognition and functional outcome in schizophrenia: implications for MATRICS. Schizophr. Res. 72, 41–51 10.1016/j.schres.2004.09.009 15531406

[B115] Groman S. M. Lee B. London E. D. Mandelkern M. A. James A. S. Feiler K. (2011). Dorsal striatal D2-like receptor availability covaries with sensitivity to positive reinforcement during discrimination learning. J. Neurosci. 31, 7291–7299 10.1523/JNEUROSCI.0363-11.2011 21593313 PMC3114883

[B116] Grottick A. J. Higgins G. A. (2000). Effect of subtype selective nicotinic compounds on attention as assessed by the five-choice serial reaction time task. Behav. Brain Res. 117, 197–208 10.1016/S0166-4328(00)00305-3 11099773

[B117] Habara T. Hamamura T. Miki M. Ohashi K. Kuroda S. (2001). M100907, a selective 5-HT(2A) receptor antagonist, attenuates phencyclidine-induced Fos expression in discrete regions of rat brain. Eur. J. Pharmacol. 417, 189–194 10.1016/S0014-2999(01)00926-8 11334850

[B118] Hajos M. Hajos-Korcsok E. Sharp T. (1999). Role of the medial prefrontal cortex in 5-HT1A receptor-induced inhibition of 5-HT neuronal activity in the rat. Br. J. Pharmacol. 126, 1741–1750 10.1038/sj.bjp.0702510 10372816 PMC1565963

[B119] Hajos M. Richards C. D. Szekely A. D. Sharp T. (1998). An electrophysiological and neuroanatomical study of the medial prefrontal cortical projection to the midbrain raphe nuclei in the rat. Neuroscience 87, 95–108 10.1016/S0306-4522(98)00157-2 9722144

[B120] Haluk D. M. Floresco S. B. (2009). Ventral striatal dopamine modulation of different forms of behavioral flexibility. Neuropsychopharmacology 34, 2041–2052 10.1038/npp.2009.21 19262467

[B121] Harder J. A. Ridley R. M. (2000). The 5-HT1A antagonist, WAY 100 635, alleviates cognitive impairments induced by dizocilpine (MK-801) in monkeys. Neuropharmacology 39, 547–552 10.1016/S0028-3908(99)00179-3 10728875

[B122] Harrison A. A. Everitt B. J. Robbins T. W. (1997). Central 5-HT depletion enhances impulsive responding without affecting the accuracy of attentional performance: interactions with dopaminergic mechanisms. Psychopharmacology (Berl). 133, 329–342 10.1007/s002130050410 9372531

[B123] Harrison A. A. Everitt B. J. Robbins T. W. (1999). Central serotonin depletion impairs both the acquisition and performance of a symmetrically reinforced go/no-go conditional visual discrimination. Behav. Brain Res. 100, 99–112 10.1016/S0166-4328(98)00117-X 10212057

[B124] Herrero J. L. Gieselmann M. A. Sanayei M. Thiele A. (2013). Attention-induced variance and noise correlation reduction in macaque V1 is mediated by NMDA receptors. Neuron 78, 729–739 10.1016/j.neuron.2013.03.029 23719166 PMC3748348

[B125] Higgins G. A. Ballard T. M. Huwyler J. Kemp J. A. Gill R. (2003a). Evaluation of the NR2B-selective NMDA receptor antagonist Ro 63-1908 on rodent behaviour: evidence for an involvement of NR2B NMDA receptors in response inhibition. Neuropharmacology 44, 324–341 10.1016/S0028-3908(02)00402-1 12604092

[B126] Higgins G. A. Enderlin M. Haman M. Fletcher P. J. (2003b). The 5-HT2A receptor antagonist M100,907 attenuates motor and “impulsive-type” behaviours produced by NMDA receptor antagonism. Psychopharmacology (Berl). 170, 309–319 10.1007/s00213-003-1549-0 12904968

[B127] Hikosaka O. Miyashita K. Miyachi S. Sakai K. Lu X. (1998). Differential roles of the frontal cortex, basal ganglia, and cerebellum in visuomotor sequence learning. Neurobiol. Learn. Mem. 70, 137–149 10.1006/nlme.1998.3844 9753593

[B128] Homayoun H. Moghaddam B. (2007). NMDA receptor hypofunction produces opposite effects on prefrontal cortex interneurons and pyramidal neurons. J. Neurosci. 27, 11496–11500 10.1523/JNEUROSCI.2213-07.2007 17959792 PMC2954603

[B129] Hondo H. Nakahara T. Nakamura K. Hirano M. Uchimura H. Tashiro N. (1995). The effect of phencyclidine on the basal and high potassium evoked extracellular GABA levels in the striatum of freely-moving rats: an *in vivo* microdialysis study. Brain Res. 671, 54–62 10.1016/0006-8993(94)01319-D 7728533

[B130] Honey G. D. Suckling J. Zelaya F. Long C. Routledge C. Jackson S. (2003). Dopaminergic drug effects on physiological connectivity in a human cortico-striato-thalamic system. Brain 126, 1767–1781 10.1093/brain/awg184 12805106 PMC3838939

[B131] Honey R. A. Honey G. D. O'Loughlin C. Sharar S. R. Kumaran D. Bullmore E. T. (2004). Acute ketamine administration alters the brain responses to executive demands in a verbal working memory task: an FMRI study. Neuropsychopharmacology 29, 1203–1214 10.1038/sj.npp.1300438 15100698 PMC3838946

[B132] Huntley G. W. Vickers J. C. Morrison J. H. (1994). Cellular and synaptic localization of NMDA and non-NMDA receptor subunits in neocortex: organizational features related to cortical circuitry, function and disease. Trends Neurosci. 17, 536–543 10.1016/0166-2236(94)90158-9 7532339

[B133] Hutson P. H. Barton C. L. Jay M. Blurton P. Burkamp F. Clarkson R. (2000). Activation of mesolimbic dopamine function by phencyclidine is enhanced by 5-HT(2C/2B) receptor antagonists: neurochemical and behavioural studies. Neuropharmacology 39, 2318–2328 10.1016/S0028-3908(00)00089-7 10974315

[B134] Ichikawa J. Ishii H. Bonaccorso S. Fowler W. L. O'Laughlin I. A. Meltzer H. Y. (2001). 5-HT(2A) and D(2) receptor blockade increases cortical DA release via 5-HT(1A) receptor activation: a possible mechanism of atypical antipsychotic-induced cortical dopamine release. J. Neurochem. 76, 1521–1531 10.1046/j.1471-4159.2001.00154.x 11238736

[B135] Jackson M. E. Homayoun H. Moghaddam B. (2004). NMDA receptor hypofunction produces concomitant firing rate potentiation and burst activity reduction in the prefrontal cortex. Proc. Natl. Acad. Sci. U.S.A. 101, 8467–8472 10.1073/pnas.0308455101 15159546 PMC420417

[B136] Jakab R. L. Goldman-Rakic P. S. (1998). 5-Hydroxytryptamine2A serotonin receptors in the primate cerebral cortex: possible site of action of hallucinogenic and antipsychotic drugs in pyramidal cell apical dendrites. Proc. Natl. Acad. Sci. U.S.A. 95, 735–740 10.1073/pnas.95.2.735 9435262 PMC18490

[B137] Jakab R. L. Goldman-Rakic P. S. (2000). Segregation of serotonin 5-HT2A and 5-HT3 receptors in inhibitory circuits of the primate cerebral cortex. J. Comp. Neurol. 417, 337–348 10.1002/(SICI)1096-9861(20000214)417:3<337::AID-CNE7>3.0.CO;2-O 10683608

[B138] Jin X. Costa R. M. (2010). Start/stop signals emerge in nigrostriatal circuits during sequence learning. Nature 466, 457–462 10.1038/nature09263 20651684 PMC3477867

[B139] Jin X. Tecuapetla F. Costa R. M. (2014). Basal ganglia subcircuits distinctively encode the parsing and concatenation of action sequences. Nat. Neurosci. 17, 423–430 10.1038/nn.3632 24464039 PMC3955116

[B140] Kellendonk C. Simpson E. H. Polan H. J. Malleret G. Vronskaya S. Winiger V. (2006). Transient and selective overexpression of dopamine D2 receptors in the striatum causes persistent abnormalities in prefrontal cortex functioning. Neuron 49, 603–615 10.1016/j.neuron.2006.01.023 16476668

[B141] Klodzinska A. Bijak M. Tokarski K. Pilc A. (2002). Group II mGlu receptor agonists inhibit behavioural and electrophysiological effects of DOI in mice. Pharmacol. Biochem. Behav. 73, 327–332 10.1016/S0091-3057(02)00845-6 12117586

[B142] Koskinen T. Ruotsalainen S. Puumala T. Lappalainen R. Koivisto E. Mannisto P. T. (2000). Activation of 5-HT2A receptors impairs response control of rats in a five-choice serial reaction time task. Neuropharmacology 39, 471–481 10.1016/S0028-3908(99)00159-8 10698013

[B143] Kuroki T. Ichikawa J. Dai J. Meltzer H. Y. (1996). R(+)-8-OH-DPAT, a 5-HT1A receptor agonist, inhibits amphetamine-induced serotonin and dopamine release in rat medial prefrontal cortex. Brain Res. 743, 357–361 10.1016/S0006-8993(96)01111-0 9017269

[B144] Lavoie B. Parent A. (1991). Serotoninergic innervation of the thalamus in the primate: an immunohistochemical study. J. Comp. Neurol. 312, 1–18 10.1002/cne.903120102 1744240

[B145] Lebedev M. A. Messinger A. Kralik J. D. Wise S. P. (2004). Representation of attended versus remembered locations in prefrontal cortex. PLoS Biol. 2:e365 10.1371/journal.pbio.0020365 15510225 PMC524249

[B146] Lee B. London E. D. Poldrack R. A. Farahi J. Nacca A. Monterosso J. R. (2009). Striatal dopamine d2/d3 receptor availability is reduced in methamphetamine dependence and is linked to impulsivity. J. Neurosci. 29, 14734–14740 10.1523/JNEUROSCI.3765-09.2009 19940168 PMC2822639

[B147] Lena I. Chessel A. Le Pen G. Krebs M. O. Garcia R. (2007). Alterations in prefrontal glutamatergic and noradrenergic systems following MK-801 administration in rats prenatally exposed to methylazoxymethanol at gestational day 17. Psychopharmacology (Berl). 192, 373–383 10.1007/s00213-007-0719-x 17279373

[B148] Le Pen G. Grottick A. J. Higgins G. A. Moreau J. L. (2003). Phencyclidine exacerbates attentional deficits in a neurodevelopmental rat model of schizophrenia. Neuropsychopharmacology 28, 1799–1809 10.1038/sj.npp.1300208 12784101

[B149] Lewis R. (2004). Should cognitive deficit be a diagnostic criterion for schizophrenia? J. Psychiatry Neurosci. 29, 102–113 15069464 PMC383342

[B150] Lillrank S. M. O'Connor W. T. Oja S. S. Ungerstedt U. (1994). Systemic phencyclidine administration is associated with increased dopamine, GABA, and 5-HIAA levels in the dorsolateral striatum of conscious rats: an *in vivo* microdialysis study. J. Neural Transm. Gen. Sect. 95, 145–155 10.1007/BF01276433 7532416

[B151] Liu S. Bubar M. J. Lanfranco M. F. Hillman G. R. Cunningham K. A. (2007). Serotonin2C receptor localization in GABA neurons of the rat medial prefrontal cortex: implications for understanding the neurobiology of addiction. Neuroscience 146, 1677–1688 10.1016/j.neuroscience.2007.02.064 17467185 PMC2913252

[B152] Lopez-Gil X. Artigas F. Adell A. (2009). Role of different monoamine receptors controlling MK-801-induced release of serotonin and glutamate in the medial prefrontal cortex: relevance for antipsychotic action. Int. J. Neuropsychopharmacol. 12, 487–499 10.1017/S1461145708009267 18752722

[B153] Lopez-Gil X. Babot Z. Amargos-Bosch M. Sunol C. Artigas F. Adell A. (2007). Clozapine and haloperidol differently suppress the MK-801-increased glutamatergic and serotonergic transmission in the medial prefrontal cortex of the rat. Neuropsychopharmacology 32, 2087–2097 10.1038/sj.npp.1301356 17356574

[B154] Lopez-Gil X. Jimenez-Sanchez L. Romon T. Campa L. Artigas F. Adell A. (2012). Importance of inter-hemispheric prefrontal connection in the effects of non-competitive NMDA receptor antagonists. Int. J. Neuropsychopharmacol. 15, 945–956 10.1017/S1461145711001064 21733285

[B155] Luck S. J. Gold J. M. (2008). The construct of attention in schizophrenia. Biol. Psychiatry 64, 34–39 10.1016/j.biopsych.2008.02.014 18374901 PMC2562029

[B156] Lustig C. Kozak R. Sarter M. Young J. W. Robbins T. W. (2013). CNTRICS final animal model task selection: control of attention. Neurosci. Biobehav. Rev. 37, 2099–2110 10.1016/j.neubiorev.2012.05.009 22683929 PMC3490036

[B157] Malhotra A. K. Pinals D. A. Weingartner H. Sirocco K. Missar C. D. Pickar D. (1996). NMDA receptor function and human cognition: the effects of ketamine in healthy volunteers. Neuropsychopharmacology 14, 301–307 10.1016/0893-133X(95)00137-3 8703299

[B158] Marek G. J. Wright R. A. Gewirtz J. C. Schoepp D. D. (2001). A major role for thalamocortical afferents in serotonergic hallucinogen receptor function in the rat neocortex. Neuroscience 105, 379–392 10.1016/S0306-4522(01)00199-3 11672605

[B159] Marek G. J. Wright R. A. Schoepp D. D. Monn J. A. Aghajanian G. K. (2000). Physiological antagonism between 5-hydroxytryptamine(2A) and group II metabotropic glutamate receptors in prefrontal cortex. J. Pharmacol. Exp. Ther. 292, 76–87 10604933

[B160] Martin P. Waters N. Schmidt C. J. Carlsson A. Carlsson M. L. (1998). Rodent data and general hypothesis: antipsychotic action exerted through 5-Ht2A receptor antagonism is dependent on increased serotonergic tone. J. Neural Transm. 105, 365–396 10.1007/s007020050064 9720968

[B161] Martin P. Waters N. Waters S. Carlsson A. Carlsson M. L. (1997). MK-801-induced hyperlocomotion: differential effects of M100907, SDZ PSD 958 and raclopride. Eur. J. Pharmacol. 335, 107–116 10.1016/S0014-2999(97)01188-6 9369362

[B162] Martin-Ruiz R. Puig M. V. Celada P. Shapiro D. A. Roth B. L. Mengod G. (2001). Control of serotonergic function in medial prefrontal cortex by serotonin-2A receptors through a glutamate-dependent mechanism. J. Neurosci. 21, 9856–9866 11739593 10.1523/JNEUROSCI.21-24-09856.2001 PMC6763049

[B163] Matsumoto M. Kanno M. Togashi H. Ueno K. Otani H. Mano Y. (2003). Involvement of GABAA receptors in the regulation of the prefrontal cortex on dopamine release in the rat dorsolateral striatum. Eur. J. Pharmacol. 482, 177–184 10.1016/j.ejphar.2003.10.003 14660020

[B164] Matsuyama S. Nei K. Tanaka C. (1996). Regulation of glutamate release via NMDA and 5-HT1A receptors in guinea pig dentate gyrus. Brain Res. 728, 175–180 10.1016/0006-8993(96)00395-2 8864479

[B165] Maura G. Raiteri M. (1996). Serotonin 5-HT1D and 5-HT1A receptors respectively mediate inhibition of glutamate release and inhibition of cyclic GMP production in rat cerebellum *in vitro*. J. Neurochem. 66, 203–209 10.1046/j.1471-4159.1996.66010203.x 8522955

[B166] Millan M. J. Dekeyne A. Gobert A. (1998). Serotonin (5-HT)2C receptors tonically inhibit dopamine (DA) and noradrenaline (NA), but not 5-HT, release in the frontal cortex *in vivo*. Neuropharmacology 37, 953–955 10.1016/S0028-3908(98)00078-1 9776391

[B167] Mirjana C. Baviera M. Invernizzi R. W. Balducci C. (2004). The serotonin 5-HT2A receptors antagonist M100907 prevents impairment in attentional performance by NMDA receptor blockade in the rat prefrontal cortex. Neuropsychopharmacology 29, 1637–1647 10.1038/sj.npp.1300479 15127084

[B168] Missale C. Nash S. R. Robinson S. W. Jaber M. Caron M. G. (1998). Dopamine receptors: from structure to function. Physiol. Rev. 78, 189–225 9457173 10.1152/physrev.1998.78.1.189

[B169] Mobini S. Chiang T. J. Ho M. Y. Bradshaw C. M. Szabadi E. (2000). Effects of central 5-hydroxytryptamine depletion on sensitivity to delayed and probabilistic reinforcement. Psychopharmacology (Berl). 152, 390–397 10.1007/s002130000542 11140331

[B170] Moghaddam B. Adams B. Verma A. Daly D. (1997). Activation of glutamatergic neurotransmission by ketamine: a novel step in the pathway from NMDA receptor blockade to dopaminergic and cognitive disruptions associated with the prefrontal cortex. J. Neurosci. 17, 2921–2927 9092613 10.1523/JNEUROSCI.17-08-02921.1997 PMC6573099

[B171] Moghaddam B. Adams B. W. (1998). Reversal of phencyclidine effects by a group II metabotropic glutamate receptor agonist in rats. Science 281, 1349–1352 10.1126/science.281.5381.1349 9721099

[B172] Morari M. O'Connor W. T. Ungerstedt U. Bianchi C. Fuxe K. (1996). Functional neuroanatomy of the nigrostriatal and striatonigral pathways as studied with dual probe microdialysis in the awake rat–II. Evidence for striatal N-methyl-D-aspartate receptor regulation of striatonigral GABAergic transmission and motor function. Neuroscience 72, 89–97 10.1016/0306-4522(95)00556-0 8730708

[B173] Morari M. O'Connor W. T. Ungerstedt U. Fuxe K. (1993). N-methyl-D-aspartic acid differentially regulates extracellular dopamine, GABA, and glutamate levels in the dorsolateral neostriatum of the halothane-anesthetized rat: an *in vivo* microdialysis study. J. Neurochem. 60, 1884–1893 10.1111/j.1471-4159.1993.tb13416.x 8097237

[B174] Morari M. O'Connor W. T. Ungerstedt U. Fuxe K. (1994). Dopamine D1 and D2 receptor antagonism differentially modulates stimulation of striatal neurotransmitter levels by N-methyl-D-aspartic acid. Eur. J. Pharmacol. 256, 23–30 10.1016/0014-2999(94)90611-4 7913045

[B175] Morris G. Arkadir D. Nevet A. Vaadia E. Bergman H. (2004). Coincident but distinct messages of midbrain dopamine and striatal tonically active neurons. Neuron 43, 133–143 10.1016/j.neuron.2004.06.012 15233923

[B176] Muir J. L. Everitt B. J. Robbins T. W. (1996). The cerebral cortex of the rat and visual attentional function: dissociable effects of mediofrontal, cingulate, anterior dorsolateral, and parietal cortex lesions on a five-choice serial reaction time task. Cereb. Cortex 6, 470–481 10.1093/cercor/6.3.470 8670672

[B177] Mukherjee J. Christian B. T. Narayanan T. K. Shi B. Mantil J. (2001). Evaluation of dopamine D-2 receptor occupancy by clozapine, risperidone, and haloperidol *in vivo* in the rodent and nonhuman primate brain using 18F-fallypride. Neuropsychopharmacology 25, 476–488 10.1016/S0893-133X(01)00251-2 11557161

[B178] Murphy E. R. Dalley J. W. Robbins T. W. (2005). Local glutamate receptor antagonism in the rat prefrontal cortex disrupts response inhibition in a visuospatial attentional task. Psychopharmacology (Berl). 179, 99–107 10.1007/s00213-004-2068-3 15678364

[B179] Navailles S. De Deurwaerdere P. (2011). Presynaptic control of serotonin on striatal dopamine function. Psychopharmacology (Berl). 213, 213–242 10.1007/s00213-010-2029-y 20953589

[B180] Neill J. C. Barnes S. Cook S. Grayson B. Idris N. F. McLean S. L. (2010). Animal models of cognitive dysfunction and negative symptoms of schizophrenia: focus on NMDA receptor antagonism. Pharmacol. Ther. 128, 419–432 10.1016/j.pharmthera.2010.07.004 20705091

[B181] Nicholson S. L. Brotchie J. M. (2002). 5-hydroxytryptamine (5-HT, serotonin) and Parkinson's disease - opportunities for novel therapeutics to reduce the problems of levodopa therapy. Eur. J. Neurol. 9(Suppl. 3), 1–6 10.1046/j.1468-1331.9.s3.1.x 12464115

[B182] Noudoost B. Chang M. H. Steinmetz N. A. Moore T. (2010). Top-down control of visual attention. Curr. Opin. Neurobiol. 20, 183–190 10.1016/j.conb.2010.02.003 20303256 PMC2901796

[B183] Pakhotin P. Bracci E. (2007). Cholinergic interneurons control the excitatory input to the striatum. J. Neurosci. 27, 391–400 10.1523/JNEUROSCI.3709-06.2007 17215400 PMC6672079

[B184] Pandey G. N. Dwivedi Y. Ren X. Rizavi H. S. Faludi G. Sarosi A. (2006). Regional distribution and relative abundance of serotonin(2c) receptors in human brain: effect of suicide. Neurochem. Res. 31, 167–176 10.1007/s11064-005-9006-6 16673176

[B185] Passetti F. Chudasama Y. Robbins T. W. (2002). The frontal cortex of the rat and visual attentional performance: dissociable functions of distinct medial prefrontal subregions. Cereb. Cortex 12, 1254–1268 10.1093/cercor/12.12.1254 12427677

[B186] Passetti F. Dalley J. W. Robbins T. W. (2003a). Double dissociation of serotonergic and dopaminergic mechanisms on attentional performance using a rodent five-choice reaction time task. Psychopharmacology (Berl). 165, 136–145 10.1007/s00213-002-1227-7 12420150

[B187] Passetti F. Levita L. Robbins T. W. (2003b). Sulpiride alleviates the attentional impairments of rats with medial prefrontal cortex lesions. Behav. Brain Res. 138, 59–69 10.1016/S0166-4328(02)00229-2 12493630

[B188] Pattij T. Janssen M. C. Vanderschuren L. J. Schoffelmeer A. N. van Gaalen M. M. (2007). Involvement of dopamine D1 and D2 receptors in the nucleus accumbens core and shell in inhibitory response control. Psychopharmacology (Berl). 191, 587–598 10.1007/s00213-006-0533-x 16972104

[B189] Pehrson A. L. Bondi C. O. Totah N. K. Moghaddam B. (2013). The influence of NMDA and GABA(A) receptors and glutamic acid decarboxylase (GAD) activity on attention. Psychopharmacology (Berl). 225, 31–39 10.1007/s00213-012-2792-z 22797703 PMC3580768

[B190] Pende M. Lanza M. Bonanno G. Raiteri M. (1993). Release of endogenous glutamic and aspartic acids from cerebrocortex synaptosomes and its modulation through activation of a gamma-aminobutyric acidB (GABAB) receptor subtype. Brain Res. 604, 325–330 10.1016/0006-8993(93)90384-Y 8096158

[B191] Perkinton M. S. Sihra T. S. (1998). Presynaptic GABA(B) receptor modulation of glutamate exocytosis from rat cerebrocortical nerve terminals: receptor decoupling by protein kinase C. J. Neurochem. 70, 1513–1522 10.1046/j.1471-4159.1998.70041513.x 9523568

[B192] Perreault M. L. Hasbi A. O'Dowd B. F. George S. R. (2011). The dopamine d1-d2 receptor heteromer in striatal medium spiny neurons: evidence for a third distinct neuronal pathway in Basal Ganglia. Front. Neuroanat. 5:31 10.3389/fnana.2011.00031 21747759 PMC3130461

[B193] Pezze M. A. Bast T. Feldon J. (2003). Significance of dopamine transmission in the rat medial prefrontal cortex for conditioned fear. Cereb. Cortex 13, 371–380 10.1093/cercor/13.4.371 12631566

[B194] Pezze M. A. Dalley J. W. Robbins T. W. (2007). Differential roles of dopamine D1 and D2 receptors in the nucleus accumbens in attentional performance on the five-choice serial reaction time task. Neuropsychopharmacology 32, 273–283 10.1038/sj.npp.1301073 16641946 PMC1877864

[B195] Pezze M. A. Dalley J. W. Robbins T. W. (2009). Remediation of attentional dysfunction in rats with lesions of the medial prefrontal cortex by intra-accumbens administration of the dopamine D(2/3) receptor antagonist sulpiride. Psychopharmacology (Berl). 202, 307–313 10.1007/s00213-008-1384-4 18985321

[B196] Plech A. Brus R. Kalbfleisch J. H. Kostrzewa R. M. (1995). Enhanced oral activity responses to intrastriatal SKF 38393 and m-CPP are attenuated by intrastriatal mianserin in neonatal 6-OHDA-lesioned rats. Psychopharmacology (Berl). 119, 466–473 10.1007/BF02245863 7480527

[B197] Pompeiano M. Palacios J. M. Mengod G. (1994). Distribution of the serotonin 5-HT2 receptor family mRNAs: comparison between 5-HT2A and 5-HT2C receptors. Brain Res. Mol. Brain Res. 23, 163–178 10.1016/0169-328X(94)90223-2 8028479

[B250] Pozzi L. Baviera M. Sacchetti G. Calcagno E. Balducci C. Invernizzi R. W. (2011). Attention deficit induced by blockade of N-methyl d-aspartate receptors in the prefrontal cortex is associated with enhanced glutamate release and cAMP response element binding protein phosphorylation: role of metabotropic glutamate receptors 2/3. Neuroscience 176, 336–348 10.1016/j.neuroscience.2010.11.060 21193020

[B198] Prado-Alcala R. A. Ruiloba M. I. Rubio L. Solana-Figueroa R. Medina C. Salado-Castillo R. (2003a). Regional infusions of serotonin into the striatum and memory consolidation. Synapse 47, 169–175 10.1002/syn.10158 12494399

[B199] Prado-Alcala R. A. Solana-Figueroa R. Galindo L. E. Medina A. C. Quirarte G. L. (2003b). Blockade of striatal 5-HT2 receptors produces retrograde amnesia in rats. Life Sci. 74, 481–488 10.1016/j.lfs.2003.06.012 14609726

[B200] Przegalinski E. Filip M. (1997). Stimulation of serotonin (5-HT)1A receptors attenuates the locomotor, but not the discriminative, effects of amphetamine and cocaine in rats. Behav. Pharmacol. 8, 699–706 10.1097/00008877-199712000-00004 9832955

[B201] Puig M. V. Celada P. Diaz-Mataix L. Artigas F. (2003). *In vivo* modulation of the activity of pyramidal neurons in the rat medial prefrontal cortex by 5-HT2A receptors: relationship to thalamocortical afferents. Cereb. Cortex 13, 870–882 10.1093/cercor/13.8.870 12853374

[B202] Puig M. V. Watakabe A. Ushimaru M. Yamamori T. Kawaguchi Y. (2010). Serotonin modulates fast-spiking interneuron and synchronous activity in the rat prefrontal cortex through 5-HT1A and 5-HT2A receptors. J. Neurosci. 30, 2211–2222 10.1523/JNEUROSCI.3335-09.2010 20147548 PMC6634052

[B203] Puumala T. Sirvio J. (1998). Changes in activities of dopamine and serotonin systems in the frontal cortex underlie poor choice accuracy and impulsivity of rats in an attention task. Neuroscience 83, 489–499 10.1016/S0306-4522(97)00392-8 9460757

[B204] Ragozzino M. E. (2002). The effects of dopamine D(1) receptor blockade in the prelimbic-infralimbic areas on behavioral flexibility. Learn. Mem. 9, 18–28 10.1101/lm.45802 11917003 PMC155930

[B205] Rasmusson A. M. Goldstein L. E. Deutch A. Y. Bunney B. S. Roth R. H. (1994). 5-HT1a agonist +/-8-OH-DPAT modulates basal and stress-induced changes in medial prefrontal cortical dopamine. Synapse 18, 218–224 10.1002/syn.890180307 7855734

[B206] Ridley R. M. Clark B. A. Durnford L. J. Baker H. F. (1993). Stimulus-bound perseveration after frontal ablations in marmosets. Neuroscience 52, 595–604 10.1016/0306-4522(93)90409-9 8450961

[B207] Robbins T. W. (1996). Dissociating executive functions of the prefrontal cortex. Philos. Trans. R. Soc. Lond. B Biol. Sci. 351, 1463–1470 discussion: 1470–1461 8941958 10.1098/rstb.1996.0131

[B208] Robbins T. W. (2002). The 5-choice serial reaction time task: behavioural pharmacology and functional neurochemistry. Psychopharmacology (Berl). 163, 362–380 10.1007/s00213-002-1154-7 12373437

[B209] Robbins T. W. (2005). Role of cortical and striatal dopamine in cognitive function, in Dopamine, eds Dunnett S. B. Bentivoglio M. Bjorklund A. (Amsterdam: Elsevier B. V.), 395–434

[B210] Robbins T. W. (2013). Optimazing the executive: neurochemical modulation of the fronto-executive “toolbox”, in Principles of Frontal Lobe Function. 2nd Edn., eds Stuss D. T. Knight R. T. (New York, NY: Oxford University Press), 55–68

[B211] Robbins T. W. Brown V. J. (1990). The role of the striatum in the mental chronometry of action: a theoretical review. Rev. Neurosci. 2, 181–214 10.1515/REVNEURO.1990.2.4.181 21561254

[B212] Rogers R. D. Baunez C. Everitt B. J. Robbins T. W. (2001). Lesions of the medial and lateral striatum in the rat produce differential deficits in attentional performance. Behav. Neurosci. 115, 799–811 10.1037/0735-7044.115.4.799 11508719

[B213] Rowland L. M. Bustillo J. R. Mullins P. G. Jung R. E. Lenroot R. Landgraf E. (2005). Effects of ketamine on anterior cingulate glutamate metabolism in healthy humans: a 4-T proton MRS study. Am. J. Psychiatry 162, 394–396 10.1176/appi.ajp.162.2.394 15677610

[B214] Saalmann Y. B. Pigarev I. N. Vidyasagar T. R. (2007). Neural mechanisms of visual attention: how top-down feedback highlights relevant locations. Science 316, 1612–1615 10.1126/science.1139140 17569863

[B215] Sakaue M. Somboonthum P. Nishihara B. Koyama Y. Hashimoto H. Baba A. (2000). Postsynaptic 5-hydroxytryptamine(1A) receptor activation increases *in vivo* dopamine release in rat prefrontal cortex. Br. J. Pharmacol. 129, 1028–1034 10.1038/sj.bjp.0703139 10696105 PMC1571922

[B216] Salamone J. D. Correa M. (2012). The mysterious motivational functions of mesolimbic dopamine. Neuron 76, 470–485 10.1016/j.neuron.2012.10.021 23141060 PMC4450094

[B217] Santana N. Bortolozzi A. Serrats J. Mengod G. Artigas F. (2004). Expression of serotonin1A and serotonin2A receptors in pyramidal and GABAergic neurons of the rat prefrontal cortex. Cereb. Cortex 14, 1100–1109 10.1093/cercor/bhh070 15115744

[B218] Sautel F. Griffon N. Sokoloff P. Schwartz J. C. Launay C. Simon P. (1995). Nafadotride, a potent preferential dopamine D3 receptor antagonist, activates locomotion in rodents. J. Pharmacol. Exp. Ther. 275, 1239–1246 8531087

[B219] Sawaguchi T. Goldman-Rakic P. S. (1991). D1 dopamine receptors in prefrontal cortex: involvement in working memory. Science 251, 947–950 10.1126/science.1825731 1825731

[B220] Scruggs J. L. Patel S. Bubser M. Deutch A. Y. (2000). DOI-Induced activation of the cortex: dependence on 5-HT2A heteroceptors on thalamocortical glutamatergic neurons. J. Neurosci. 20, 8846–8852 11102493 10.1523/JNEUROSCI.20-23-08846.2000 PMC6773058

[B221] Scruggs J. L. Schmidt D. Deutch A. Y. (2003). The hallucinogen 1-[2,5-dimethoxy-4-iodophenyl]-2-aminopropane (DOI) increases cortical extracellular glutamate levels in rats. Neurosci. Lett. 346, 137–140 10.1016/S0304-3940(03)00547-0 12853103

[B222] Seamans J. K. Yang C. R. (2004). The principal features and mechanisms of dopamine modulation in the prefrontal cortex. Prog. Neurobiol. 74, 1–58 10.1016/j.pneurobio.2004.05.006 15381316

[B223] Seeman P. Ulpian C. (1983). Neuroleptics have identical potencies in human brain limbic and putamen regions. Eur. J. Pharmacol. 94, 145–148 10.1016/0014-2999(83)90452-1 6140171

[B224] Shallice T. (1982). Specific impairments of planning. Philos. Trans. R. Soc. Lond. B Biol. Sci. 298, 199–209 10.1098/rstb.1982.0082 6125971

[B225] Shi W. X. Zhang X. X. (2003). Dendritic glutamate-induced bursting in the prefrontal cortex: further characterization and effects of phencyclidine. J. Pharmacol. Exp. Ther. 305, 680–687 10.1124/jpet.102.046359 12606677

[B226] Smith J. W. Gastambide F. Gilmour G. Dix S. Foss J. Lloyd K. (2011). A comparison of the effects of ketamine and phencyclidine with other antagonists of the NMDA receptor in rodent assays of attention and working memory. Psychopharmacology (Berl). 217, 255–269 10.1007/s00213-011-2277-5 21484239

[B227] Sokoloff P. Giros B. Martres M. P. Bouthenet M. L. Schwartz J. C. (1990). Molecular cloning and characterization of a novel dopamine receptor (D3) as a target for neuroleptics. Nature 347, 146–151 10.1038/347146a0 1975644

[B228] Soubrié P. (1986). Reconciling the role of central serotonin neurons in human and animal behaviour. Behav. Brain Sci. 9, 319–364 10.1017/S0140525X00022871

[B229] Steinbusch H. W. M. (1984). Serotonin-immunoreactive neurons and their projections in the CNS, in Handbook of Chemical Neuroanatomy, eds Bjorklund A. Hokfelt T. Kuhar M. J. (Amsterdam: Elsevier Science), 68–125

[B230] Surmeier D. J. Shen W. Day M. Gertler T. Chan S. Tian X. (2010). The role of dopamine in modulating the structure and function of striatal circuits. Prog. Brain Res. 183, 149–167 10.1016/S0079-6123(10)83008-0 20696319 PMC4431764

[B231] Swanson C. J. Schoepp D. D. (2002). The group II metabotropic glutamate receptor agonist (-)-2-oxa-4-aminobicyclo[3.1.0.]hexane-4,6-dicarboxylate (LY379268) and clozapine reverse phencyclidine-induced behaviors in monoamine-depleted rats. J. Pharmacol. Exp. Ther. 303, 919–927 10.1124/jpet.102.038422 12438510

[B232] Thomson A. M. (2000). Molecular frequency filters at central synapses. Prog. Neurobiol. 62, 159–196 10.1016/S0301-0082(00)00008-3 10828382

[B233] Trantham-Davidson H. Neely L. C. Lavin A. Seamans J. K. (2004). Mechanisms underlying differential D1 versus D2 dopamine receptor regulation of inhibition in prefrontal cortex. J. Neurosci. 24, 10652–10659 10.1523/JNEUROSCI.3179-04.2004 15564581 PMC5509068

[B234] Tritsch N. X. Sabatini B. L. (2012). Dopaminergic modulation of synaptic transmission in cortex and striatum. Neuron 76, 33–50 10.1016/j.neuron.2012.09.023 23040805 PMC4386589

[B235] Ungerstedt U. (1971). Postsynaptic supersensitivity after 6-hydroxy-dopamine induced degeneration of the nigro-striatal dopamine system. Acta Physiol. Scand. Suppl. 367, 69–93 4332693 10.1111/j.1365-201x.1971.tb11000.x

[B236] Varty G. B. Bakshi V. P. Geyer M. A. (1999). M100907, a serotonin 5-HT2A receptor antagonist and putative antipsychotic, blocks dizocilpine-induced prepulse inhibition deficits in Sprague-Dawley and Wistar rats. Neuropsychopharmacology 20, 311–321 10.1016/S0893-133X(98)00072-4 10088132

[B237] Wang M. Yang Y. Wang C. J. Gamo N. J. Jin L. E. Mazer J. A. (2013). NMDA receptors subserve persistent neuronal firing during working memory in dorsolateral prefrontal cortex. Neuron 77, 736–749 10.1016/j.neuron.2012.12.032 23439125 PMC3584418

[B238] Wang X.-J. (2013). The prefrontal cortex as a quintessential “cognitive type” neural circuit: working memory and decision making, in Principles of Frontal Lobe Function. 2nd Edn., eds Stuss D. T. Knight R. T. (New York, NY: Oxford University Press), 226–248

[B239] Ward R. P. Dorsa D. M. (1996). Colocalization of serotonin receptor subtypes 5-HT2A, 5-HT2C, and 5-HT6 with neuropeptides in rat striatum. J. Comp. Neurol. 370, 405–414 8799865 10.1002/(SICI)1096-9861(19960701)370:3<405::AID-CNE10>3.0.CO;2-R

[B240] Wedzony K. Mackowiak M. Zajaczkowski W. Fijal K. Chocyk A. Czyrak A. (2000). WAY 100135, an antagonist of 5-HT1A serotonin receptors, attenuates psychotomimetic effects of MK-801. Neuropsychopharmacology 23, 547–559 10.1016/S0893-133X(00)00150-0 11027920

[B241] Wilkinson R. T. (1963). Interaction of noise with knowledge of results and sleep deprivation. J. Exp. Psychol. 66, 332–337 10.1037/h0044161 14051850

[B242] Winstanley C. A. Chudasama Y. Dalley J. W. Theobald D. E. Glennon J. C. Robbins T. W. (2003a). Intra-prefrontal 8-OH-DPAT and M100907 improve visuospatial attention and decrease impulsivity on the five-choice serial reaction time task in rats. Psychopharmacology (Berl). 167, 304–314 10.1007/s00213-003-1398-x 12677356

[B243] Winstanley C. A. Theobald D. E. Dalley J. W. Glennon J. C. Robbins T. W. (2004a). 5-HT2A and 5-HT2C receptor antagonists have opposing effects on a measure of impulsivity: interactions with global 5-HT depletion. Psychopharmacology (Berl). 176, 376–385 10.1007/s00213-004-1884-9 15232674

[B244] Yamada H. Matsumoto N. Kimura M. (2004). Tonically active neurons in the primate caudate nucleus and putamen differentially encode instructed motivational outcomes of action. J. Neurosci. 24, 3500–3510 10.1523/JNEUROSCI.0068-04.2004 15071097 PMC6729748

[B245] Yerkes R. M. Dodson J. D. (1908). The relation of strength of stimulus to rapidity of habit-formation. J. Comp. Neurol. Psychol. 18, 459–482 10.1002/cne.920180503

[B246] Yonezawa Y. Kuroki T. Kawahara T. Tashiro N. Uchimura H. (1998). Involvement of gamma-aminobutyric acid neurotransmission in phencyclidine-induced dopamine release in the medial prefrontal cortex. Eur. J. Pharmacol. 341, 45–56 10.1016/S0014-2999(97)01435-0 9489855

[B247] Young A. M. Bradford H. F. (1993). N-methyl-D-aspartate releases gamma-aminobutyric acid from rat striatum *in vivo*: a microdialysis study using a novel preloading method. J. Neurochem. 60, 487–492 10.1111/j.1471-4159.1993.tb03176.x 8093478

[B248] Zahrt J. Taylor J. R. Mathew R. G. Arnsten A. F. (1997). Supranormal stimulation of D1 dopamine receptors in the rodent prefrontal cortex impairs spatial working memory performance. J. Neurosci. 17, 8528–8535 9334425 10.1523/JNEUROSCI.17-21-08528.1997 PMC6573725

[B249] Zhai Y. George C. A. Zhai J. Nisenbaum E. S. Johnson M. P. Nisenbaum L. K. (2003). Group II metabotropic glutamate receptor modulation of DOI-induced c-fos mRNA and excitatory responses in the cerebral cortex. Neuropsychopharmacology 28, 45–52 10.1038/sj.npp.1300013 12496939

